# Analysis of Effectiveness and Psychological Techniques Implemented in mHealth Solutions for Middle-Aged and Elderly Adults with Type 2 Diabetes: A Narrative Review of the Literature

**DOI:** 10.3390/jcm10122701

**Published:** 2021-06-18

**Authors:** Julia Vázquez-de Sebastián, Andreea Ciudin, Carmina Castellano-Tejedor

**Affiliations:** 1 RE-FiT Barcelona Research Group, Vall d’Hebron Institute of Research & Parc Sanitari Pere Virgili, 08023 Barcelona, Spain; julia.vazquez@vhir.org; 2 Institut de Recerca Vall d’Hebron, Universitat Autònoma de Barcelona (VHIR-UAB), 08035 Barcelona, Spain; 3 CIBER de Diabetes y Enfermedades Metabólicas Asociadas, Instituto de Salud Carlos III, Spain & Endocrinology Department, University Hospital Vall d’Hebron, 08035 Barcelona, Spain; 4 GIES Research Group, Basic Psychology Department, Autonomous University of Barcelona, Bellaterra, 08192 Barcelona, Spain

**Keywords:** diabetes, mHealth, elderly, adherence, self-management, psychological techniques

## Abstract

Background: in diabetes, multiple mHealth solutions were produced and implemented for self-management behaviors. However, little research on the effectiveness of psychological techniques implemented within these mHealth solutions was carried out, and even less with the elderly population where technological barriers might exist. Reliable evidence generated through a comprehensive evaluation of mHealth interventions may accelerate its growth for successful long-term implementation and to help to experience mHealth benefits in an enhanced way in all ages. Objective: this study aimed to review mHealth solutions for diabetes self-management in older adults (adherence to treatments and glycemic control) by analyzing the effectiveness of specific psychological techniques implemented. Methods: a narrative review was conducted following preferred reporting items for systematic reviews and meta-analyses (PRISMA) guidelines. PubMed (Medline) and American Psychological Association (APA) PsycInfo databases were searched for published papers that addressed eHealth solutions’ effectiveness for diabetes self-management. Studies in English, Spanish, and/or German of any design were screened, with no time constraints regarding the year of publication. A qualitative analysis of the selected papers was conducted in several steps. Results: this review found 38 studies setting up and analyzing mHealth solutions for older adults. Most research showed improvements in HbA1c, self-management behaviors, and medication adherence in T2DM patients post intervention. However, different mid-to-long term effects were found across studies, specifically concerning the maintenance and adherence to healthy behaviors. The most employed psychological framework was CBT, including techniques such as self-monitoring of outcome behaviors (mostly targeting glycemia measurements and healthy habits as physical activity and/or diet), tailored motivational feedback from medical staff, and psychoeducation or health coaches. The most successful mHealth intervention combined the feature of tailored feedback messages, interactive communication with healthcare professionals, and multifaceted functions. Conclusions: there is a lack of elaborate and detailed information in the literature regarding the factors considered in the design and development of mHealth solutions used as interventions for T2DM self-management in the elderly. Documentation and inclusion of such vital information will foster a transparent and shared decision-making process that will ultimately lead to the development of useful and user-friendly self-management apps that can enhance the quality of life for diabetes patients. Further research adapting mHealth solutions to older adults’ sensory deficits is necessary.

## 1. Introduction

The term mHealth was introduced in 2003 due to the vast expansion of mobile communication technologies. mHealth is the only Information and Communication Technologies (ICT) solution able to involve the transmission, storage, and receipt of multimedia files in a patient-healthcare provider synchronized manner [1]. The Global Observatory for eHealth (GOe) defined mHealth as “*medical and public health practice supported by mobile devices, such as mobile phones, patient monitoring devices, personal digital assistants (PDAs), and other wireless devices”* [2]. According to WHO (2008), mHealth can be defined as “*mobile computing, medical sensor, and communications technologies for health care*” [2,3]. mHealth employs various features, including SMS text messages, emails, phone calls, and mobile phone apps. Therefore, this technology’s potential use is evident, both for the general population and clinical samples, since it is employed to improve healthy behaviors and self-care in many medical conditions. Hence, mHealth offers interesting opportunities to transform every step of patients’ management journey, such as booking appointments, consultation with healthcare professionals, information acquisition, monitoring, and learning about treatments, drugs, and self-management, among other potentialities. However, the trustworthiness and robustness of mHealth to facilitate safe and cost-efficient care are being questioned because of the lack of strong evidence [4]. This situation may trigger reluctance in investing in and developing mHealth-related policies in organizations and countries.

Type 2 Diabetes (T2D) is a chronic non-communicable disease very prevalent in older adults, caused by a complex interaction of multiple factors, of which unhealthy lifestyles such as sedentary behavior and nutritional habits play a significant role, especially in the elderly [5]. According to the International Diabetes Foundation (IDF), 253.4 million people ≥65 years will be living with diabetes in 2045 [6,7]. Besides, people >65 years old are expected to grow from an estimated 524 million in 2010 to nearly 1.5 billion in 2050, especially in developing countries [8,9,10]. This situation brings a significant economic burden to healthcare, reaching more than 160 billion euros (181 billion USD) in Europe, the second highest among all IDF regions, since the 60–69 age group the one with the largest expenditure on people with T2D, followed by 70–79 years and 50–59 years, successively [8,9,10].

To face this situation, efforts should be placed to improve diabetes self-management and support. Limited medication adherence and poor compliance with treatment are the main barriers to the successful management of T2D [11]. In this sense, only 50% of patients reach their HbA1c target [12]. It is crucial because poor glycemic control is associated with many diverse complications, such as diabetic retinopathy, nephropathy, neuropathy, cardiovascular disease, other disabilities, and a decrease in quality of life (QoL), therefore increasing the risk of comorbidities and death [13,14]. According to evidence-based guidelines, the seven self-care key behaviors for patients with diabetes are (1) eating healthy, (2) being active, (3) monitoring, (4) taking medications, (5) solving problems, (6) healthy coping, and (7) reducing risks [15,16]. Considering that traditional outpatient consultations cannot provide timely support and feedback regularly, mHealth solutions could be a more ecological and efficient approach to educate and facilitate these actions [17]. Mobile apps can collect, record, monitor, transmit, and analyze data anytime and anywhere. Thanks to these potentialities, health apps could foster and reinforce self-management behaviors and medication adherence in diabetic users, even also without in-office training [18]. Additionally, these apps represent a great tool to improve not only self-management, but also communication in terms of empowerment and enrichment between elderly patients and healthcare professionals [19], especially at a time when, due to the global COVID-19 pandemic, remote health care became a priority. A systematic review revealed that diabetes apps are one of the most common disease-specific apps developed and commercialized worldwide, probably because they allow for the comprehensive assessment and support of the above referred seven self-care key behaviors [20]. In this sense, these apps could serve insulin, medication and diet recording, data export and communication, and weight management [21,22,23,24]. 

Recent RCT trials [24,25] found no significant differences in using different apps to improve glycemic control (HbA1c levels). However, these studies do not provide detailed information on whether their designs were based on clinical guidelines and recommendations, including reinforcing educational and supportive aspects [24,25]. Therefore, despite this vast market holding promising beneficial effects for users, 44% of them abandon health apps just one month after being downloaded [26].

Different reasons could explain this lack of engagement. In many cases, no solid theoretical frameworks support the development and implementation of different techniques to engage potential mHealth app users and seem not to be integrated into the ecology of the doctor-patient relationship [27]. Besides, most commercial apps were not evaluated using scientific methods, and these apps tend not to be grounded explicitly in theories of health behavior fostering adherence and motivation among users [28]. When it comes to acceptance and usability requirements for older adults with diabetes, this is especially relevant because, in this sample, several barriers could be found. For instance, the lack of technological skills [29,30,31], availability and access to the internet [32], differences in motivational aspects [33], and also functional and sensory limitations hampering the use of these technologies. A systematic review of all currently available diabetes apps for iOS and Android revealed that usability of diabetes apps for patients aged ≥50 was moderate to good when applied mainly to apps offering a small range of functions. However, complex multifunctional apps performed considerably worse in terms of usability [29]. Thus, building and maintaining sustained motivation to adhere elderly users to mHealth is still an unresolved problem.

Considering all these reasons, health apps need to be evaluated on (a) their potential to start and/or support healthy lifestyles, (b) their consistency with evidence-based practices and solid psychological theories related to motivation and behavior change, and (c) their effectiveness in improving HbA1c levels (mean HbA1c reduction) as the standard measure of average glycemic control predicting diabetes complications. Therefore, this research aimed to systematically review mHealth solutions for T2D self-management in older adults by analyzing the effectiveness of specific psychological techniques implemented. 

## 2. Materials and Methods

### 2.1. Study Design

A systematic search of relevant literature was conducted following Preferred Reporting Items for Systematic reviews and Meta-Analyses (PRISMA) guidelines [34]. 

### 2.2. Study Selection

This research sought to identify empirical studies of any design describing mHealth non-pharmacological interventions (cognitive-behavioral, psychological/educational) based on apps aimed at fostering and/or maintaining self-management of T2D in older adults. 

mHealth involves the use of mobile devices’ technological potentialities (i.e., general packet radio service (GPRS), third and fourth generation mobile telecommunications (3G and 4G systems), a global positioning system (GPS), and Bluetooth technology) for reporting health information, monitoring clinical signs, and enabling direct supervision and instruction. In the present review, we adhered to Bashshur and colleagues’ definition of mHealth [35]. These authors agree that mHealth can be understood as an umbrella term encompassing the preceding domains of telemedicine, telehealth, and eHealth. Still, they also highlighted that mHealth includes further possibilities, such as smart device-based mHealth interventions. That is to say, the use of a smartphone, tablet, or peripheral device and their features (such as high computing power, advanced functions using apps, use of sensors, fast network speed, or the interactive multitouch screen to deliver health care services) for clinical support, health worker support, remote data collection, and helpline [35].

We conceptualized the non-pharmacological interventions applied to diabetes as any type of program/action addressing self-management of T2D, including medication adherence and proper blood glucose monitoring. Also, interventions to foster adherence to healthy lifestyles (e.g., regular physical activity and healthy diet) can be included in combination with previous actions. 

Old age includes the period of life after youth and middle age. There is no defined age cut-off point, as this is context-dependent and, as WHO recognizes, this period is not defined by years. However, the United Nations assume that a cut-off lower age can be 65 years. Despite this fact, the scientific literature in this field of study reveals certain laxity with these criteria and often the inclusion of younger samples is considered correct. We decided to establish as an inclusion criterion a lower cut-off for age 50±10, with no upper limit due to a high proportion of manuscripts based on older adult populations which also included subsamples of younger participants.

Considering all of the above, inclusion criteria were the following: (1) studies carried out with patients diagnosed with T2DM (lower cut-off for age 50±10, no upper limit); (2) interventions had to use app-based tools that are accessible via mobile phone or tablet to aid self-management of T2D; (3) and reported outcomes should be any one of the following: clinical data for either self-management of T2D, medication adherence-related outcome, or change in self-management behavior. To be eligible for this review, research describing interventions aimed at fostering or reinforcing healthy lifestyles must be combined necessarily with any type of action directed to improve or favor T2D self-management, such as glycemic control and medication adherence.

Studies were excluded if they did not include detailed information on the mHealth non-pharmacological interventions carried out, no data on the effectiveness of the solution were provided (e.g., study protocols), just usability, satisfaction, or acceptance results were detailed, or the intervention only targeted health care providers. Other exclusions included those designed from the scope of telemedicine, telehealth, and/or eHealth (not mainly based on health apps). Review studies were screened to identify additional studies to be included in this research. English, Spanish, and German publications were considered hence articles in any other language were excluded. No time constraints were set.

### 2.3. Information Sources

MEDLINE (using PubMed) and APA PsycInfo databases were searched. The gray literature was searched through the following databases: MedEdPortal, Proquest: dissertations, and OpenGrey. The reference list of retrieved papers and relevant systematic reviews were also searched.

### 2.4. Search Terms

Table 1 shows search terms and strings employed with their corresponding Booleans to locate relevant studies for this review.

### 2.5. Data Extraction

Study citations were imported and compiled into the reference management software (Mendeley) for selection. The screening and selection of studies were conducted by two independent researchers individually (JV & CCT). Both researchers reviewed all titles and abstracts selected by the database queries. JV manually removed duplicates. In case of disagreement, a third reviewer with expertise in the field was consulted and made the final decision on inclusion (AC). The second stage involved reviewing the full-text versions of the selected articles by both reviewers (JV & CCT) from the selected abstracts. The snowballing method was conducted on the reference lists of relevant articles. Controversial studies and problems were compared and discussed with JV, CCT, and AC. To optimize the data extraction and charting process, an Excel file was created to collate the following information pertinent to the aims of this research, including the following headings: author(s) and year of publication, design, sample size and target group, intervention (type and specific techniques), length of intervention, objectives, and main results of the study. The results of this extraction process were then synthesized and interpreted.

The quality of the included studies was evaluated by indicating which of them followed (total or partially) the Mobile Health Evidence Reporting and Assessment (mERA) Checklist [36] or the RE-AIM Framework [37]. These checklists were developed from the Consolidated Standards of Reporting Trials of Electronic and Mobile Health Applications and onLine TeleHealth (CONSORT-EHEALTH), and were extended to a wide range of mHealth interventions in different healthcare settings [38]. It consists of 2 separate assessments: (1) essential criteria for mHealth, which comprise 16 items that identify the content, context, and technical features to ensure the quality and generalizability of mHealth research, and (2) methodological criteria, which include 26 items that are based on existing study design and study-reporting guidelines. 

## 3. Results

### 3.1. Synthesis of Search Results

**Figure 1:**
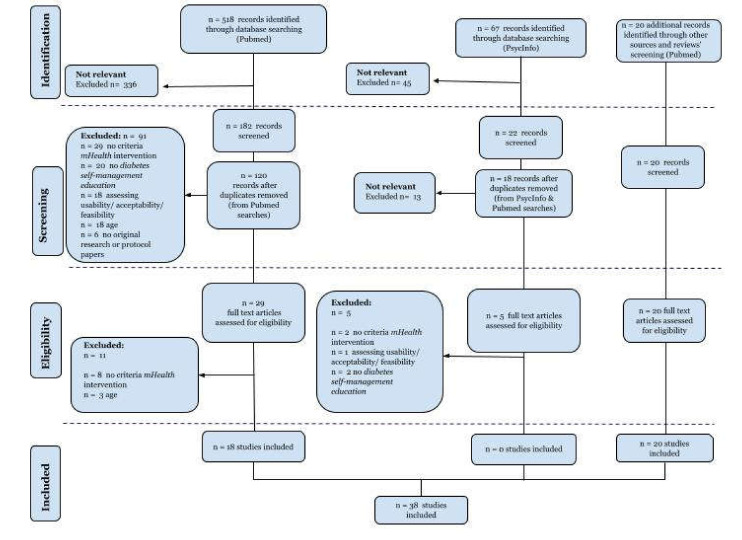
Flow diagram of study selection.

The initial search strategy according to search strings noted in Table 1 produced 739 articles; 518 were retrieved from PubMed, 67 from PsycInfo, 154 from screened lists of references of 25 relevant to the topic review studies, identified while conducting the initial PubMed search, and 2 additional studies were found while searching for “related paper” suggested by PubMed. After excluding irrelevant records and removing duplicates, 54 full-text articles were assessed for eligibility. The remaining articles were full text reviewed, and after excluding records not matching inclusion criteria, a collection of 38 articles was selected to be included and analyzed in-depth in this review. Figure 1 displays the flow of the review search.

### 3.2. Synthesis of Study Characteristics and Outcome Measures

The main characteristics of the 38 studies included in this review are shown in detail in Supplementary Material Table S1.

Concerning the geographical origin of research included in this review, 11 studies were from the European Continent (3 from the United Kingdom, 3 from Norway, 2 from Finland, 2 from Germany, and 1 from Poland), 13 were from the American Continent (11 from USA and 2 from Canada), 12 from Asia (3 from South Korea, 3 from China, and 1 each from Saudi Arabia, Lebanon and Syria, Singapore, India, Japan, and Taiwan), and 1 each from Africa (RD Congo) and Oceania (Australia). 

All articles included were conducted in urban or suburban areas of developed countries, except one study conducted in a rural area [39]. Four articles specifically targeted vulnerable and low socioeconomic strata populations [22,40,41,42], and one study was carried out with a particular population composed of older US veterans [43].

Thirty out of 38 articles included T2DM patients (*n =* 4336, T2DM patients) analyzed as a single study sample or divided into different groups depending on the study design. Four studies were carried out including a sample composed of T1 and T2 diabetic patients (*n =* 292) [22,44,45,46], and one study was carried out with a sample of unspecified type of diabetes (*n =* 40) [47]. The three remaining articles described research conducted with a sample of patients with increased cardiovascular risk (*n =* 53) [48], multiple chronic conditions (*n =* 1860) [49], and overweight patients with T2DM and hypertension (*n =* 111) [50]; all of them focused on the integrated management of non-communicable diseases (NCD). The mean sample size from all included articles was 186 patients, ranging from 3–2,400 patients.

The different settings chosen to recruit the sample were general tertiary hospitals (6 studies) [39,46,51,52,53,54], primary care settings (18 studies) [22,40,41,42,49,55,56,57,58,59,60,61,62,63,64,65,66,67], outpatient endocrinology clinics from different healthcare centers (7 studies) [43,44,48,68,69,70,71], samples from both primary care and other health centers (4 studies) [45,50,72,73], other healthcare settings (not specified) [47], specific health facilities such as a medical center of a military area [74], and a clinic electronic health database from a research center [75].

The mean age of patients across 33 out of the 38 included articles was 57.96 years old (SD 9.81). Mean age and the standard deviation was not specified in 5 articles; only reporting age was cut off as inclusion criteria (41–50 years [74]; >40 years [40] and >50 years group [68]), providing median values (67.97 years old) [71] or just indicating mean (59.6) and age range (46–71), but not SD [58]. 

All articles included in this review were intervention studies (average length of the interventions 6.58 months, range 1–20 months) with different samples and designs, collecting a series of pre/post measures. Among them, different mechanisms for controlling biases such as randomization, masking, and the inclusion of control groups were established. Twenty-two out of 38 were designed as randomized clinical trials (RCTs). Among them, blinding (doctor blinded) was used in just one study [48]. The rest of the included studies were not blinded but included different conditions: such as 2 crossover 2-arms [48,68]; two with 2 parallel-arms (no control group) [40,62]; two studies included 3-arms [72,73]; 3-arms cross-over [54]; 4-arms [64], and one study was carried out including 5-arms [43]. Twelve out of the 38 selected articles did not include control groups [22,39,41,45,57,58,59,66,69,70], and one study was a nonrandomized, quasi-experimental design [51]. Finally, one study was conceived as a retrospective, propensity score-matched design [75].

A total of 33 studies measured glycated hemoglobin (HbA1c) reduction as an outcome variable indicating the effectiveness of the different interventions. Of these, two studies used blood glucose (mg/dL) as the only clinical measure [22,54] collected in the research. Nineteen studies used change (%, pre/post intervention) in HbA1c as primary outcome [41,42,43,44,49,52,53,56,57,59,60,61,64,69,72,73,74,75,76], three as a secondary outcome [45,48,55], and ten studies measured this variable not specifying their role in the statistical analyses [39,40,46,47,50,51,58,62,71], or just as a baseline measure with no post intervention HbA1c values [68]. Other clinical outcomes collected were blood pressure (BP), weight, waist circumference, body mass index (BMI), and lipid levels as measures to assess the efficacy of the different interventions implemented. Among studies considering the change in HbA1c (%) as a primary outcome, 10 articles reported significant differences between the intervention and the control group [53,64,75,76], and one of them indicated that these improvements in the intervention group were not determined by age (younger/older 55 years; 45–64 years) [76]. On the contrary, five studies evidenced HbA1c improvements in all groups with no statistically significant differences between intervention and control groups [41,44,49,72,73]. Among studies considering HbA1c as a secondary outcome, all reported improvement pre/post intervention (35,36,41–44,46,47, 51,54,58,67). However, only 8 studies pointed out that these improvements reach statistical significance (4–44,46,47,67) in the same sample (41,43,44) or between groups (42,46,47,67). 

Besides glycemia reduction, additional measures of interventions’ effectiveness were self-efficacy related to diabetes self-management behaviors, mainly based on medication adherence, increasing physical activity, improving dietary patterns, and reducing smoking and alcohol consumption [22,40,42,43,49,53,56,61,66,67,68,69,71,72,73,77,78]. 

A total of 11 studies reported insulin dosage adjustments and changes in medication prescriptions [44,46,48,53,55,57,61,65,69,70,71]. Statistically significant improvements in medication use (metformin or insulin dose) [48,55,57,70], a higher proportion of medication titrated or adjustments performed by the reference healthcare professionals [61,65,71], and more frequent glucose self-monitoring behaviors were described in intervention groups, correlating with higher rates of HbA1c reduction [69] compared to that of control groups. No statistical significance differences within or between-groups concerning changes in daily insulin dosages or frequency of endocrinology appointments were observed in one study [44]. Other studies reached no conclusive results concerning between-groups comparison related to changes in medication and insulin dose [46,53].

Mental health status was collected as a secondary outcome in 15 articles. Except one study [73], all studies showed improvements regarding depressive symptoms [49,66,72], anxiety [22,42,44,55,56,67,70,71,73], and impact of diabetes on health-related quality of life and distress [45,58,64]. However, these between-group effects were only statistically significant in some research [22,42,44,55,56,67,70,71,73]. 

Nine articles evidenced between-groups differences with a more significant improvement of self-management behaviors based on the practice of healthy habits among participants in the intervention group [39,46,54,55,67,69,72,73,74]. However, these benefits were not sustained at 3–9 months follow-up [67], nor at 4–8 months follow-up, except for an improvement in diet and reduced smoking [39]. No group-effect was shown in three studies [53,56,68], but when the tool is used with high-intensity, between-differences in self-efficacy and exercise were elicited [54]. Furthermore, greater adherence to healthy behaviors (i.e., higher locomotion) was associated with a more significant reduction in HbA1c [43]. The length of the intervention showed controversial results, since at the beginning of its implementation effects seemed quicker and higher, but this pattern of constant improvement is not maintained in subsequent months [39,42,75]. Additionally, specific diabetes-health education as a key action to improved self-management and self-efficacy was assessed in four articles administering a specific diabetes knowledge test [46,54,71,74], all of them reporting better results post intervention. 

Usability, acceptance, and user’s satisfaction with the mHealth solution were analyzed in 17 articles through patients’ and stakeholders’ experience perceptions using surveys, interviews, or data directly collected from the mHealth system. 

Older patients (>62 years old) revealed a higher use of the proposed mHealth solution compared to younger patients [52,72]. Patients described the system as a helpful tool; however, some disadvantages such as costs for internet connection and time needed for getting used to the application were outlined as main concerns related to usability for both users, patients, and the medical team [47].

Results showed satisfactory clinical levels of acceptance both among the group of patients and among the professionals surveyed [46,48,51,53,55,57,59,62,66,68,71], and one research pointed out that adding different motivational features to the mHealth solution did not influence acceptance results [70]. 

When analyzing patients’ satisfaction related to the mHealth solution, a reduction in HbA1c levels was observed in the more satisfied patients, contrary to what happened with those less satisfied [52]. When statistically analyzed, satisfaction with the tool was shown to be significant in some studies [45,61].

### 3.3. Type of Interventions & Psychological Techniques

Most of the mHealth solutions were based on apps [22,40,49,51,52,54,61,66,68,70], a combination of app and peripheral devices [43,46,53,56,57,59,60,72,73], or apps and web-based platforms [39,41,42,58,64,65,76]. A total of 10 studies used a combination of apps, web-based platforms, and peripheral devices [44,45,47,48,55,67,69,71,74,75], and one study was conducted with a web-portal physician communication and the use of peripheral devices connected to patients’ mobile phones [50].

In all, 14 studies explicitly drew on existing psychological theories to guide the mHealth intervention design [42,43,44,54,56,58,59,60,66,67,68,72,73,74]. All of them were built on cognitive-behavioral theories (CBT) [42,44,59,60,64,65,66,67,74,76], and specifically the transtheoretical model of stages of change and problem-solving approaches [72,73]. A total of three studies combined CBT with self-determination theory (SDT) [56] or acceptance and commitment therapy (ACT) [58] and gamification principles [43]. A minority of research was solely based on SDT [68] or other approaches, such as the social-cognitive theory combined with the Fogg Behavior Model (FBM) [54]. The majority of the research did not explain the theoretical framework employed to design and build the mHealth solution [22,39,40,41,45,46,47,48,49,50,51,52,53,55,57,61,62,69,70,71,75].

To synthesize the different psychological techniques employed in the different mHealth studied solutions, the classification of BC techniques proposed by Michie et al. (2013) [79] was used, combined with the specific techniques described within each study. In summary, 16 psychological techniques were identified: psychoeducation, goal setting, action planning, rewards, behavior substitution rehearsal, self-monitoring of outcome behaviors, tailored feedback from medical staff on outcome behavior, automatic feedback on outcome behavior, behavior substitution rehearsal, social support (general, practical, emotional), pharmacological support, MI techniques, problem-solving, counseling, stress regulation/relaxation techniques, reminders.

Most of the studies (*n =* 37, 97%) focused on self-monitoring of outcome behaviors are mainly based on T2DM parameters (specifically, glycemia) and healthy habits such as improving or fostering the regular practice of physical exercise, following dietary recommendations concerning daily calories and nutrients intake, and adherence to medication [22,39,40,42,43,44,45,46,47,48,49,50,51,52,53,54,55,56,57,58,59,60,61,62,64,65,66,67,68,69,70,71,72,73,74,75,76]. In this sense, a total of 31 studies included psychoeducation features and/or health coaches (either virtual health coaches or a health professional receiving, monitoring, and analyzing participants’ responses) [40,41,42,43,44,45,47,48,49,50,51,52,53,54,56,58,59,60,61,62,64,65,66,68,69,71,72,73,74,75,76]. Similarly, 13 studies set alerts to improve medication adherence [22,41,42,43,46,49,50,52,54,56,62,70,71], 31 studies provided motivational encouragement to strengthen the patient’s self-efficacy [39,40,41,42,44,45,46,47,48,49,50,52,53,54,57,58,60,61,62,64,65,66,67,69,71,73,74,75,76], 4 studies employed social support through chat services to enhance intervention [43,54,69,74], 3 studies provided a decision support system for an action plan made by doctors, pharmacists, or nurses [46,56,64], and 1 intervention included stress management [58].

Table 2 summarizes psychological frameworks and techniques implemented in each mHealth solution from different articles included in this review. 

## 4. Discussion

### 4.1. Discussion of Main Findings

This narrative review aimed to identify mHealth solutions for older adults with T2DM and analyze its effectiveness to support self-management (medication and glycemic control) and adherence to healthy lifestyle recommendations. Further, we sought to examine the different psychological frameworks that guided the design of the solution and the different techniques embedded in its functioning.

Thirty-eight studies with a total sample of 4,336 older adults with T2DM and a mean age of 57.96 ± 9.81 were identified. This low number of research papers with older adults’ population contrasts with the great proliferation of studies in the same field, which exist for young people or young adults, mainly diagnosed with T1DM [80,81]. In most clinical trials and research studies, the elderly population is underrepresented [82]. Comorbidities, difficulty traveling to research sites, and a reluctance to undergo demanding research procedures are some of the reasons elderly patients do not participate in different research designs. The greater health risks and possible lack of support from relatives are also common difficulties to enroll aged individuals in very demanding trials. In this sense, most studies propose specific exclusion criteria, such as sensory and visual deficits, or cognitive impairment, all of which are very common in elderly populations with diabetes. All these reasons make it difficult to draw generalizable conclusions to this subsample from studies with the general adult population. 

It is also worth noting that most older adults’ research was conducted including individuals of a wide age range. Although there is some consensus to consider the age cut at 65, the reality makes it very difficult to recruit these subsamples, and frequently, these studies include participants below this cut-off point.

Different barriers and facilitators were described in research with older adult populations, particularly in research involving mHealth solutions. One of the main barriers is the resistance to the adoption of new technologies for low ability, accessibility, costs, or usability [83]. Also, age-related decline in cognitive flexibility, processing speed, and other cognitive functions in old age can make it challenging to adopt and manage these solutions well, which could mean a low adherence [63]. Other aspects to be carefully considered when developing mHealth solutions for the elderly are short-set ups and onboarding processes, and user-friendly and graphic visual cues facilitating the understandability of the mHealth solution [29,30,31,84]. Acceptance and usability requirements of the elderly diabetic patients were insufficiently considered despite these growing populations representing a large target group that could benefit from diabetes apps [85]. Our review observed that only 17 studies report specific data on usability, satisfaction, and acceptance.

Despite these difficulties, according to this review, almost all studies showed promising results concerning the different solutions tested. Specifically, most interventions resulted in better glycemic control than usual care, with statistically significant decreases that some research also demonstrated were maintained at mid–long term [60,71]. However, we barely found research carrying out follow-up assessments sufficiently long in time to ensure sustainability in the use of the tested technology and the durability of the beneficial effects on health and self-management of patients’ diabetes. Future studies should be designed to have more extended follow-up periods to test whether positive 1-year intervention effects can be sustained among larger samples of older adults with T2DM patients despite low-usage attrition or dropout [86].

Findings of this review confirmed that, in general terms, self-management education through mHealth was effective in increasing patients’ knowledge of T2DM, healthy lifestyle, and medication management, and consequently, significant increases in self-efficacy were observed [42,51,65,68,69,70]. However, these benefits were not sustained at mid-long term follow-ups [42,51,63,65,68,69,70], with few exceptions for some particular health behaviors [35]. Interestingly, different psychoeducation forms were embedded in the other solutions (either through automatic feedback, pre-established recommendations, or real-time interaction with healthcare professionals). Although psychoeducation has on numerous occasions demonstrated its potential beneficial effect to promote healthy behaviors and/or prevent health complications [87,88,89], the sustainability of its impact over time was questioned, and a more comprehensive psychological framework could enhance its impact and facilitate sustainability over time. 

Outcomes concerning design frameworks and techniques embedded in the different studied technologies revealed low efforts to detail psychological principles underlying the app’s different functionalities. Twenty-one studies did not specify the reference conceptual framework to develop and design the mHealth solution and were not explicitly attached to any taxonomy of techniques to describe the different features implemented. Thus, most investigations only vaguely described the application’s operation without offering much detail that facilitates understanding the dynamics and, therefore, the app’s actions and effects on its users’ behavior.

Different authors have highlighted the need to use available scientific knowledge to support these interventions’ development [90]. Diabetes self-management is recognized as the cornerstone of overall diabetes management. mHealth programs favoring self-management for T2DM patients can successfully improve patient health behaviors and health-related outcomes. Theories can help to specify key determinants of the target behaviors and specific behavior change strategies required to arrive at the desired health outcomes [91]. Only in this way will it be possible to increase the likelihood of behavior change towards a healthier goal. One of the conceptual frameworks that accumulated the most empirical evidence over the years in relation to usefulness in promoting behavior change is the cognitive-behavioral approach [92,93,94]. Precisely, 14 of 38 studies base their premises on this conceptual framework, despite not delving too deeply into its descriptions. However, most research provides no solid theoretical frameworks supporting the development and implementation of different techniques to engage potential diabetes app users. 

This happens with most commercial apps (not specifically for older adult population), which tend not to be grounded explicitly in theories of health behavior, so it is unknown which app features are particularly effective and encourage sustained app usage is still unclear. Similarly, behavior change techniques (BCTs) combined with other frameworks, such as gamification and or motivational interviewing principles, are promising approaches to improve user engagement [95,96,97], but few health apps currently employ these premises and there is a wide variation in the operationalization and final use of BCTs, which may limit potential to improve health outcomes [98].

Apart from the theoretical frame of reference, different authors tried to identify key elements that should be considered when developing mHealth solutions. Mayberry et al. [99] identified different key recommendations for the design and research of mobile and Internet interventions for disadvantaged and vulnerable persons with diabetes. These elements were access issues, program design concerning the human element, understanding the targeted users and using more agile science focusing on comparative effectiveness/superiority trials, and pragmatic trials and cost analyses rather than efficacy trials with a treatment-as-usual control group. No studies based on these premises were identified in our review. The use of adaptive study designs is crucial to address the need for continued quality improvement and rapid iteration in this field. 

### 4.2. Weaknesses and Strengths of Studies Included in This Review

To our knowledge, this is the first review focused on older adults that analyzed the relationship between the characteristics of mHealth-enabled T2DM self-management and the clinical and behavioral outcomes, considering and analyzing the psychological framework as a basis for the design of such health apps. However, several limitations of the included articles should be considered when interpreting results. 

A scarce number of investigations were carried out with a homogeneous sample of T2DM older adult samples. Most of them included participants of a very wide age range and included different health conditions (e.g., other NCDs). Only 5 out of the 38 studies specifically targeted the older adult population [43,66,70,71,76], and two studies made a specific analysis stratified by age group [72,74]. Besides, criteria to include participants and to consider poorly controlled HbA1c are very varied across studies (values of HbA1c ranging from 6 to 7.5). Similarly, outcome measures for assessing the effectiveness of different interventions and self-efficacy in terms of T2DM self-management behaviors were also very different between investigations (e.g., Diabetes Management Self-Efficacy Scale—DMSES, Short Form 36 (SF-36) Health Survey, the Health Education Impact Questionnaire—heiQ). Most importantly, the main weakness identified was the observed heterogeneities in study designs and in relation to the intervention and control group features. This made it difficult to draw conclusions since there might be great variability in modulating variables that could influence the effectiveness of the mHealth solution. mHealth technologies can help older adults to improve their diabetes management as it does in younger patients, and to structure their daily routine despite their disease. However, due to the special needs and characteristics of this sample population, no generalization can be performed derived from heterogeneous studied samples in terms of age. Older patients have proved their ability to use these tools [66] showing even more intensive use than younger ones and taking advantage of mHealth to even empower communication with professionals [19,91]. For all this, these apps will most likely be a highly demanded health care strategy in post-COVID times. However, these apps must be tailored to the requirements of older adults.

In this sense, no study reported the mHealth literacy of the study sample. Sensitivity to mHealth literacy is especially important in the design of digital interventions and their content, as both are prevalent barriers to understanding and acting on health information. There is a high proportion of low mHealth literate individuals among older adults [100,101], and this might involve struggles with evaluating and trusting online health information, thereby often relying on verbal communication about their health instead, which makes direct feedback from healthcare professionals a key complement of such solutions. Further studies in this field should be more explicit about the process used to develop and/or adapt intervention content and technical functionality for T2DM elderly patients in terms of mHealth literacy, usability, satisfaction, and acceptance. Besides, more studies in low-/middle-income countries are needed since huge differences in literacy and access to new technologies could exist.

Finally, language was restricted to English, Spanish, and German, which reduces the diversity of studies analyzed. Also, most of the studies included were conducted in developed countries, which may lead to limited global generalizability. Regional discrepancies, clinical and social practices, and health care systems and policies may not be generalizable to other regions.

One positive remark is that, although they were a minority, three studies followed (total or partially) the Mobile Health Evidence Reporting and Assessment (mERA) Checklist [36] or the RE-AIM Framework [37] developed from the Consolidated Standards of Reporting Trials of Electronic and Mobile Health Applications and onLine TeleHealth (CONSORT-EHEALTH) [38] to design and develop the mHealth proposed solution. It is highly recommended to follow these guidelines to guarantee not only the quality of the technology, but also its assessment and replication of research and standardize evaluation reports of web-based and mobile health interventions.

### 4.3. Implication of Policy Making and Further Research

Considering that T2D represents a big burden for both the healthcare system and patients bearing this chronic condition, this review provided evidence that health apps aimed at fostering self-management and promoting behavior change could improve T2D management and reduce the risks of secondary complications, and eventually, mortality. There is increasing interest in comparing benefits of mHealth approaches. Questions remain to be addressed about the values of diverse mHealth methods and psychological techniques implemented. To promote mHealth interventions of self-management effectively and efficiently, more clinical studies are warranted to detect the relationship between the specific intervention pattern and outcomes. In addition, patients’ compliance with self-management interventions should be examined in the future. Finally, particular characteristics of the older adults’ population should be carefully considered since different barriers and facilitators were identified and should be incorporated from the initial steps concerning the design of such interventions. Thus, links with the generalizability; it is necessary to determine whether mHealth-based self-management methods should be tailored to age groups, cultural contexts, or need to be extended to include support from health care personnel and more long-term economic evaluation needs to be done. 

## 5. Conclusions

Effective treatment of T2DM requires careful self-management. With the ongoing development of mobile technologies and the scarcity of health care resources, mHealth–based self-management became a useful treatment for T2DM, and its effectiveness was assessed in many trials. However, there is a paucity of comprehensive summaries of the studies carried out with older adults’ population and testing mHealth solutions with a solid psychological framework supporting techniques embedded in the app. 

This review demonstrated that despite promising results in terms of self-management and adherence to treatment and healthy lifestyle recommendations, theory-driven interventions are still scarce despite being the most claimed and recommended in evidence-based guidelines. Some psychological techniques were proved to be effective in the self-management of chronic illness [93,94,95] (although there are still several gaps in the applied knowledge concerning their specific implementation and effects in older adults’ samples). Only 14 out of 38 articles were explicit in terms of which theoretical framework and psychological techniques were employed to design the mHealth intervention and to implement it. This research was mainly based on CBT alone or combined with SDT, ACT, or gamification principles proving consistency with evidence-based practices related to behavior change.

As mHealth technologies grow, emerging applications of the technologies will enable life-changing uses for the elderly population. The application of mHealth has the potential to improve health outcomes and change the course of healthcare as it is provided today, potentially reducing socioeconomic and human costs. However, more in-depth studies in the older adults’ population with T2DM are required to validate these potentialities and provide more conclusive evidence. 

## Figures and Tables

**Table 1:** Search terms used in PubMed and PsycInfo.

Search Number	PubMed Search String
1	(((diabet*[Title/Abstract]) AND adherence[Title/Abstract]) AND education*[Title/Abstract]) AND older adults[Title/Abstract]
2	(((diabet*[Title/Abstract]) AND adherence[Title/Abstract]) AND management*[Title/Abstract]) AND elderly[Title/Abstract]
3	(((diabet*[Title/Abstract]) AND adherence[Title/Abstract]) AND self*[Title/Abstract]) AND elderly[Title/Abstract]
4	(((diabet*[Title/Abstract]) AND adherence[Title/Abstract]) AND mHealth[Title/Abstract]) AND elderly[Title/Abstract]
5	(((diabet*[Title/Abstract]) AND e-blister[Title/Abstract])
6	(((diabet*[Title/Abstract]) AND mhealth[Title/Abstract]) AND older adults[Title/Abstract]
**Search Number**	**PsycInfo Search String**
1	diabetes AND adherence AND education AND older adults
2	diabetes AND adherence AND management AND elderly
3	diabetes AND adherence AND self AND elderly
4	diabetes AND adherence AND mHealth AND elderly
5	diabetes AND mHealth AND older adults
6	diabetes AND mHealth OR pill dispenser

**Table 2:** Psychological framework and specific techniques implemented in mHealth solution.

Author(s) & Year of Publication	mHealth Solution	Psychological Framework	Modules/Main Features of the mHealth Solution	Specific Techniques (Based on Michie et al. (2013) [79]
Alanzi et al., 2018 [74]	SANAD System (app + web portal + peripheral device)	CBT	Three modules: (1) glucose management, (2) social network (educational), and (3) behavior change module.	Psychoeducation, Tailored feedback from medical staff on outcome behavior, Automatic feedback on behavior, Behavior substitution, Rehearsal, Self-monitoring of outcome behavior, Social support general, practical, emotional
Baron et al., 2016 [44]	Mobile Telehealth (App + web portal + peripheral devices)	CBT	Patients received feedback from mobile SW (colored graphics) and nurses, supporting out-of-range clinical readings, insulin titration, and education calls on lifestyle changes and T2DM medication adherence.	Psychoeducation, Tailored feedback from medical staff on outcome behavior, Automatic feedback on behavior, Behavior substitution, Rehearsal, Self-monitoring of outcome behavior
Bovbjerg et al., 2017 [22]	mHealth app	n.e.	Technical training and support providing interactive biometric data and alerts.	Automatic feedback on behavior, Self-monitoring of outcome behavior, Reminders
Bramwell et al., 2019 [51]	Health2Sync (app)	n.e.	Standard insulin titration advice from a diabetes educator.	Psychoeducation, Self-monitoring of outcome behavior
Brath et al., 2013 [48]	Medication Adherence measurement system (mAms) (app + web + e-blisters)	n.e.	Calls from a coordinator to patients with poor adherence to increase adherence and motivation	Psychoeducation, Tailored feedback on outcome behavior, Self-monitoring of outcome behavior, Pharmacological support
Doocy et al., 2017 [40]	Sana Telehealth Platform (app)	n.e.	Personally controlled health record and informational printouts for patients on prescriptions and lifestyle behaviors.	Psychoeducation, Tailored feedback from medical staff on outcome behavior, Self-monitoring of outcome behavior
Dugas et al., 2018 [43]	DiaSocial app (app + Fitbit one)	CBT with gamification principles	Four conditions with different features embedded in the app: (1) individual user, (2) user + clinician, (3) user + peers (other users), (4) user + clinician + peers.	Psychoeducation, Rewards, Goal-setting, Behavioral rehearsal, Self-monitoring of outcome behavior, Tailored feedback from medical staff on outcome behavior, Automatic feedback on behavior, Social support general, practical, emotional, Reminders
Holmen et al., 2014 [72]	Few touch app (FTA) (device + app)	CBT combined with the transtheoretical model of stages of change & the problem- solving model	Two conditions: (1) the FTA system provided the user with a diabetes diary app designed to increase self-management through awareness, an overview of relevant factors, and symbols such as smiling faces and color codes in the app. (2) FTA + health counseling (as a booster for the first 4 months)	Psychoeducation, Self-monitoring of outcome behavior, Automatic feedback on behavior MI techniques, Problem-solving, Counseling
Hunt et al., 2014 [68]	Diabetes Buddy^®^ (Apple iPad app)	SDT	Self-management behaviors tracked and reviewed through a visual representation of how day-to-day activities affect outcomes (monitoring BGL diet, exercise, medication).	Psychoeducation, Self-monitoring of outcome behavior, Automatic feedback on behavior MI techniques
Kardas et al., 2016 [55]	COMMODITY12 system	n.e.	Tracking of T2DM self-management	Self-monitoring of outcome behavior, Automatic feedback on behavior
Karhula et al., 2015 [56]	Mobile phone-based health coaching program, supported by the Remote Patient Monitoring system (app + peripheral devices)	CBT SDT	Health coaches called patients every 4 to 6 weeks, and patients were encouraged to self-monitor their weight, BP, BGL, and steps once per week.	Action planning, Goal setting, MI techniques, Self-monitoring of outcome behavior, Automatic feedback on behavior, Tailored feedback from medical staff on outcome behavior (health coach), Reminders
Kim et al., 2014 [52]	Mobile Smartcare, version 1.0.7	n.e.	Warning messages, clinician feedback, and tailored recommendations to the patient an average of once per week (exercise & diet)	Psychoeducation, Self-monitoring of outcome behavior, Automatic feedback on behavior, Tailored feedback from medical staff on outcome behavior, MI techniques, Reminders
Kim et al., 2016 [69]	Patient-centered smartphone-based diabetes care system (PSDCS) (app + web + peripheral devices)	n.e.	Four modules: (1) glucose (feedback, alarm messages, recommendations), (2) diet (records of daily dietary intake, total calories, and nutrients intake) (3) physical activity (tracks activity and energy expenditure calculator, exercise video-clips), and (4) Social Network System (patients post their thoughts, opinions, and tips for diabetes self-care). PSDCS also contained diabetes self-management educational material about managing various diabetes-related conditions and specific situations.	Psychoeducation, Goal setting, Self-monitoring of outcome behavior, Automatic feedback on behavior, Tailored feedback from medical staff on outcome behavior, Social support general, practical, emotional
Koot et al., 2019 [59]	Glyco app (app + peripheral devices)	CBT	Lifestyle management program: lessons about self-management and BG monitoring with a health coaching feature.	Psychoeducation (Health coach), MI techniques, Automatic feedback on behavior
Larsen et al., 2010 [57]	App + peripheral devices	n.e.	Electronic diary with BG self-management features. The nurse provided general advice, motivation and assisted with technical problems.	Self-monitoring of outcome behavior, Tailored feedback from medical staff on outcome behavior, MI techniques
Li et al., 2020 [75]	App + web platform + data sharing cloud platform + peripheral devices	n.e.	Automatic and clinicians’ health advice (calls) for T2DM self-management (hypoglycemia and abnormal BGL, medication, diet, exercise). Patients record (pictures, descriptions) their meals and get clinicians’ feedback. Patients also have access to educational information (articles, videos, and attractive posters).	Psychoeducation, Self-monitoring of outcome behavior, Tailored feedback from medical staff on outcome behavior, MI techniques, Problem-solving
Mora et al., 2017 [45]	Accu-Chek Connect diabetes management system (app + web portal + peripheral device)	n.e.	System use, treatment changes, and recommendations (adjustments in medication, lifestyle/behavioral counseling, address skill deficit, and address nonadherence) through calls and visits.	Psychoeducation, Self-monitoring of outcome behavior, Tailored feedback from medical staff on outcome behavior
Nes et al., 2012 [58]	Few Touch Application (FTA) (app + web-based)	ACT CBT	Participants recorded their health behaviors and FBG daily on a web-based diary. A therapist had immediate access to submitted diaries and used the situational information to formulate personalized feedback. Patients have access to audio files with mindfulness and relaxation exercises.	Psychoeducation, Goal setting, Self-monitoring of outcome behavior, Tailored feedback from medical staff on outcome behavior, Automatic feedback on outcome behavior, Stress regulation/Relaxation techniques
Orsama et al., 2013 [60]	Monica app (app + peripheral devices)	CBT	Diabetes lifestyle self-management promotion program involving remote patient reporting and automated feedback.	Psychoeducation, Goal setting, Self-monitoring of outcome behavior, Tailored feedback from medical staff on outcome behavior, Automatic feedback on outcome behavior, MI techniques, Problem-solving
Prabhakaran et al., 2018 [49]	mWellcare (app)	n.e.	Self-management DSR generated based on patients collected data. The nurse provides lifestyle advice using prompts of the DSR. SMS service reminders (to take medication and attend follow-up visits).	Psychoeducation, Self-monitoring of outcome behavior, Tailored feedback from medical staff on outcome behavior Automatic feedback on outcome behavior, Reminders
Quinn et al., 2008 [61]	WellDoc’s Diabetes Management SW (app)	n.e.	Patients received personalized real-time feedback messages regarding BG readings. When problematic readings are detected, an email with specific educational material is sent.	Psychoeducation, Self-monitoring of outcome behavior, Tailored feedback from medical staff on outcome behavior, Automatic feedback on outcome behavior
Quinn et al., 2011 [64]	Mobile diabetes SW (app + web portal)	CBT	Three interventions: (1) Coach (2) Coach PCP Portal (3) Coach PCP Portal with CPDS self-management patient coaching system and provider decision support. Patients received automated, real-time educational, behavioral, and motivational messaging in response to individually analyzed data.	Psychoeducation, Self-monitoring of outcome behavior, Tailored feedback from medical staff on outcome behavior, Automatic feedback on outcome behavior, MI techniques
Quinn et al., 2016 [76]	Mobile diabetes SW (app + web portal)	CBT	Automated, real-time messages that were educational, behavioral, motivational, and specific to the data. The diabetes educators could supplement it with messages based on longitudinal diabetes self-care data trends.	Psychoeducation, Self-monitoring of outcome behavior, Tailored feedback from medical staff on outcome behavior, Automatic feedback on outcome behavior, MI techniques
Quinn et al., 2014 [65]	Mobile diabetes SW (app + web portal)	CBT	The coaching system involved patients using mobile phones to record information about their diabetes self-management. Patients received real-time (automatic) and personalized coaching feedback consistent with their treatment plans. Physicians could review patient-recorded data accessible through the provider Internet portal and received quarterly facsimile reports, including diabetes treatment recommendations.	Psychoeducation, Self-monitoring of outcome behavior, Tailored feedback from medical staff on outcome behavior, Automatic feedback on outcome behavior, MI techniques
Quinn et al., 2015 [66]	WellDoc’s Diabetes Management SW (app)	CBT	Patients enter diabetes self-care healthy data, and the system sends automated messages in addition to personalized messages to a web portal containing educational and motivational elements. The web portal also included health records that patients were encouraged to update (laboratory values, eye examinations, foot screenings, results from provider visits), a learning library, and a historical logbook.	Psychoeducation, Self-monitoring of outcome behavior, Tailored feedback from medical staff on outcome behavior, Automatic feedback on outcome behavior, MI techniques
Sittig et al., 2020 [54]	CapABILITY (app) Interactive Health Communication Application (IHCA)	Social Cognitive Theory (SCT) focused on self-efficacy. Fogg Behavior Model (FBM)	Patients interact and receive spark and facilitators educational messages (media and text) about diet, exercise, and self-management (e.g., medication adherence, BGL).	Psychoeducation, Self-monitoring of outcome behavior, Tailored feedback from medical staff on outcome behavior, Automatic feedback on outcome behavior, Reminders Social support
Steinert et al., 2017 [70]	My Therapy (app)	n.e.	Patients transfer self-management data. The app acts as a reminder of taking medication, measurements, or physical activity.	Self-monitoring of outcome behavior, Reminders
Sun et al., 2019 [71]	mHealth management app (app + medical server + peripheral devices)	n.e.	Patients uploaded the glucometer data to the app transmitted to the medical server. The medical team sent medical advice and reminders to monitor their BG by message or telephonically. The dietitian received daily dietary records and once per month sent nutritional recommendations. Patients sent information about physical activity and were provided guidance related to aerobic and resistance-based exercises.	Psychoeducation, Reminders, Self-monitoring of outcome behavior, Tailored feedback from medical staff on outcome behavior, Automatic feedback on outcome behavior.
Takenga et al., 2014 [47]	Mobil Diab System (web portals + app + peripheral device)	n.e.	Patients enter diabetes-related data, received in real-time by medical care providers. Therapy plans, instructions, and recommendations sent by the doctor from the doctor portal are received directly in the app. Patients without smartphones can still get these doctors’ feedback in the protected patients’ portal through their email addresses or SMS. Doctors receive messages from their patients directly in the web portal. An SMS is automatically generated and sent to the treating physician for emergency cases and gives direct instructions to the patient.	Psychoeducation, Goal setting, Planning, Self-monitoring of outcome behavior, Tailored feedback from medical staff on outcome behavior, Automatic feedback on outcome behavior
Torbjornsen et al., 2014 [73]	The Few Touch Application (FTA) diabetes diary with or without health counseling(app + peripheral devices)	CBT (The Transtheoretical Model + The Problem Solving Model)	Five components for data management: (1) BGL, (2) food habits, (3) physical activity, (4) personal goal-setting system, and (5) general diabetes information look-up system. Patients receive calls from nurses, and they can contact via text messages.	Psychoeducation, Goal setting, Planning, Self-monitoring of outcome behavior, Tailored feedback from medical staff on outcome behavior, Automatic feedback on outcome behavior, MI techniques, Problem-solving, Health counseling
Turner et al., 2009 [62]	t+ Diabetes app (app + peripheral devices)	n.e.	Provides real-time data transmission and feedback to patients on their mobile phone through (1) transmission of blood glucose test results and real-time feedback of trends to the mobile phone; (2) an electronic patient diary with the facility to record insulin doses; and (3) a facility to transmit blood pressure results and weight. Immediate feedback is delivered with summaries and charts to make self-management decisions. Nurses and clinicians send automated alerts and reminders. Nurses encourage patients to follow insulin titration recommendations, advised by the GP.	Self-monitoring of outcome behavior, Tailored feedback from medical staff on outcome behavior, Automatic feedback on outcome behavior, Psychoeducation, Reminders
Waki et al., 2014 [53]	DialBetics System (app + peripheral devices)	n.e.	Patients measure their health data, and if readings are abnormal, these are reported to a physician that interacts with the patient. If reading is abnormal or missed, the medical staff can email the patient to measure their data and involve the specialists and experts if their help was required. The participants can contact the nurse by smartphone or email for equipment failures, technical questions, or in response to alerts. For inquiries related to their health status, they consult their GP. Patients send voice/text messages about behaviors and receive immediate advice on lifestyle modification. Patients can view their measurement data and graphic outputs of their diet and exercise history.	Psychoeducation, Self-monitoring of outcome behavior, Tailored feedback from medical staff on outcome behavior, Automatic feedback on outcome behavior, MI techniques
Wayne & Ritvo, 2014 [41]	Connected Health and Wellness Platform (CHWP) Health Coach app 1.0 version	n.e.	The app tracks health behaviors and self-monitors health data. Wellness plans, collaboratively created in multiple interactions focused on exercise instruction and reviews of electronic monitoring entries. The program prompts to engage in health behaviors and reports on satiety levels.	Psychoeducation, Goal setting, Self-monitoring of outcome behavior, Behavior substitution rehearsal, Tailored feedback from medical staff on outcome behavior, Automatic feedback on outcome behavior, Reminders
Wayne et al., 2015 [42]	Connected Wellness Platform (CWP) (app)	CBT	Platform supported patients in health-related goal setting and progress monitoring. Participants could track key metrics, including diabetes self-care, health data, and mood. They could communicate with their health coach at any time via messaging, scheduled phone contact, and in-person meetings. Based on patient goals, clinicians guide healthy lifestyle choices while providing support when clients diverge from intended health goals and routines. All attended weekly team meetings to discuss behavior theory applications in specific strategies for each participant. Intervention and control group assisted an exercise education program that featured exercise prescription, monitoring, and adherence support.	Psychoeducation, Goal setting, Self-monitoring of outcome behavior, Tailored feedback from medical staff on outcome behavior, Automatic feedback on outcome behavior, MI techniques, Reminders
Yoo et al., 2009 [50]	The Ubiquitous Chronic Disease Care (UCDC) system (web Portal + peripheral devices)	n.e.	Participants received SMS and reminders concerning practice and recording different behaviors and disease self-management. Immediate feedback, messages of encouragement, and recommendations (automatic and from physicians) are received regularly and displayed on the website.	Psychoeducation, Goal setting, Self-monitoring of outcome behavior, Tailored feedback from medical staff on outcome behavior, Automatic feedback on outcome behavior, MI techniques, Reminders
Young et al., 2020 [67]	MyFitnessPal mobile app Health Record (app + peripheral devices + connectors + Health Record system)	CBT	The tracking device generated real-time information about healthy data. Participants can log and track nutritional consumption in the mobile app. In-person or telephonic technical support is available. Participants, primary care providers, and nurse health coaches view trends on their smartphones or computers.	Goal setting, Self-monitoring of outcome behavior, Tailored feedback from medical staff on outcome behavior, Automatic feedback on outcome behavior, MI techniques
Yu et al., 2020 [39]	Intergenerational Mobile Technology Opportunities Program (IMTOP) (app + web database)	n.e.	Participants can record their health data, medication adherence and mood. Recorded data is transmitted to the web-based database.	Self-monitoring of outcome behavior, Tailored feedback from medical staff on outcome behavior, Automatic feedback on outcome behavior
Zhou et al., 2016 [46]	Welltang app (app + peripheral device)	n.e.	A virtual educator for diabetes and a virtual endocrinologist for clinicians, facilitating the integration of diabetes care among existing resources. Patients-clinician communication comprised patients receiving advice from the study team, usually within the day based on their entered questions (BGL, target goals, individualized medication regimens). The database also triggers alerts for missed readings. Patients received an electronic action plan per month as pre-visit summaries for physician office visits.	Action planning, Goal setting, Self-monitoring of outcome behavior, Tailored feedback from medical staff on outcome behavior, Automatic feedback on outcome behavior, MI techniques, Reminders

Note: ACT: Acceptance and commitment therapy; BG: Blood Glucose; BGL: Blood Glucose Levels; CBT: Cognitive-behavioral techniques; CPDS: Coach Portal Decision-support; DSR: Decision Support Recommendations; FBG: Fasting Blood Glucose; FBM: Fogg Behavior Model; GP: General Practitioner; HC: Health Coaches; MI: Motivational interviewing; n.e.: not specified; SCT: Social Cognitive Therapy; SDT: Self-determination theory; SW: Software; T2DM: Type 2 Diabetes Mellitus; PCP: Primary Care Providers.

## Data Availability

Not applicable.

## Supplementary Materials

The following are available online at https://www.mdpi.com/article/10.3390/jcm10122701/s1, Table S1: Summary of articles included in the review.
