# Supplementary material for: Analysis of Effectiveness and Psychological Techniques Implemented in mHealth Solutions for Middle-Aged and Elderly Adults with Type 2 Diabetes: A Narrative Review of the Literature
Source: J Clin Med. 2021 Jun 18;10(12):2701. doi: 10.3390/jcm10122701 (PMC8235068; doi:10.3390/jcm10122701)
Supplement: Supplementary file 1 [file jcm-10-02701-s001.zip › jcm-1259583-supplementary.pdf]

Table S1. Summary of articles included in the review.

Author (s) & Year	Design	Sample size; target group	Intervention: Type and specific techniques	Length of the intervention	Objective(s) of the study	Main results	Following Consort eHealth guidelines (32-34)
Alanzi et al. 2018 (70)	Randomized Controlled Trial (RCT)	N=20. T2DM Age groups: 18-40 years (n=17); 41-50 years (n=3).	mHealth technology/tool: Saudi Arabia Networking Aiding Diabetes (SANAD) system<br><br>Tool purpose/ functioning: three principal modules; 1) diabetes management (SMDMM), 2) social networking (SNM) and 3) cognitive-behavioral therapy module (CBT-M). The SMDMM was developed to collect blood glucose data wirelessly from Smartphone using Bluetooth technologies from the glucose sensors; the SNM acts as an improvement educational module for the SMDMM, and the CBT-M designed on the Smartphone platform is utilized for complementary behavioral change by patients who require CBT intervention.	6 months	To examine the effect of the SANAD system on the improvement of glycaemic control, health awareness and self-efficacy.	Mean HbA1c decreased significantly after the SANAD intervention process. Age significantly predicted change in HbA1c (%) in the intervention group, with older age (i.e., 41–50 years) associated with higher HbA1c (%) at 6 months, as compared with baseline values.<br>The mean diabetes knowledge score prior to the intervention rose significantly afterward. Age, gender, and educational level were not related to increased diabetes knowledge in each study group.<br>The mean self-efficacy score prior to the intervention rose significantly afterward. Age, gender, and educational level were not related to increased self-efficacy in each study group.	
**Baron et al. 2016 (40)**	**RCT**	**N=81 poorly controlled type 1 and T2DM patients (intervention group, n=45; TAU, n=36) Age groups: intervention group 58.2±13.6 years old; TAU 55.8±13.8 years old.**	**mHealth technology/tool: Mobile Telehealth (MTH) equipment includes BG meter, BP monitor, mobile-phone, and Bluetooth cradle.**<br><br>**Tool purpose/ functioning: The mobile-phone SW allowed participants to store and transmit diabetes-related data (BG and BP readings, time since last meal,**	**9 months**	**To determine the effects of MTH on HbA1c and other clinical and patient-reported outcomes in insulin-requiring people with diabetes**	**The Group by Time effect revealed MTH did not significantly influence HbA1c, but p values were borderline significant for BP (p=0.054) and mental dimensions from HRQoL assessment (p=0.057). Examination of effect sizes**	

			level of physical activity performed that day, insulin dose, and weight) to an MTH platform (self-monitoring, mobile-phone data transmissions, graphical and nurse-initiated feedback, and educational calls). Participants were recommended to continue to follow their regular BG and BP self-monitoring routine (a minimum of one clinical reading per month was recommended for those not prescribed self-monitoring BP). Color-coded graphical feedback on the data recorded could be accessed through the mobile-phone menu and was automatically displayed following each data transfer. The MTH nurses accessed the data transmitted to the server via Web portal also accessible to MTH patients. In addition to providing feedback on out-of-range clinical readings (as needed) and education on lifestyle changes (six weekly educational calls), the MTH nurses supported insulin titration; if a more substantial medication review was required, participants were recommended to make an appointment with their diabetes specialist nurse.			and 95% CI for mean group differences at 9 months supported the existence of a protective effect of MTH on mental HRQoL as well as depression. None of the other measured outcomes were found to be affected by the MTH intervention.
Bovbjerg et al. 2017 (19)	Pre-post pilot trial	N=33 (24 completed) Low-income population with diabetes; three also had chronic obstructive pulmonary disease, one also had asthma, and three also had coronary artery disease. Age group: 54.8±12.2 years old.	<u>mHealth technology/tool</u>: mHealth application (app)<br><br><u>Tool purpose/ functioning:</u> to connect high-cost, high-utilizing Medicaid patients with diabetes to four nurse case managers at a large multispecialty clinic in the Pacific Northwest. The app installed in a tablet includes text messaging; displays of real-time, interactive biometric data, including BGL, weight, and BP; and provides	6-12 months	To test a new patient-friendly mHealth solution that incorporates multiple communication and connectivity features, including biometric monitoring, in a low-income population with complex chronic diseases.	The quality of care provided to participating patients improved over the course of the project. Patients' self-rated wellness improved. No changes were observed in physical health, social relationships, or environment domains (data not shown). Patients' mental health improved. Patients' self-efficacy to manage their

			**alerts for out-of-range biometric readings or urgent messages. The app automatically records self-monitored BGL via a cellular-enabled glucose meter, which was given to patients along with the tablet.**			**chronic disease(s) did not change over the course of this project, although a bimodal distribution was observed, with some patients reporting extremely high levels of self-efficacy and others reporting very low levels (data not shown). Self-monitored BGL overall did not change by a clinically significant amount. However, this overall statistic masks some large changes that occurred at the individual level.**
Bramwell et al. 2019 (47)	Quasi-experimental pre-post pilot study	N=92; TAU (n=50) or Health2Sync (H2S) (n=42) treatment group. T2DM patients Age group: Intervention group=56±11 years old; control group=64±13 years old.	mHealth technology/tool: the Health2Sync app<br><br>Tool purpose/ functioning: Patients in the intervention arm actively logged their BGL readings (pre and post meals) in the app on their mobile phone during the week, and these appear on a dashboard on the Credentialed Diabetes Educator's (CDE) computer; later reviewed by the CDE on a designated day, at which point the patient and CDE would communicate through the built-in chat function in the app. Patients were individually provided with BGL targets by their endocrinologist in the clinic before starting the treatment. Titration occurred between initial and follow-up appointments, which were scheduled approximately three months apart.	3 months	To examine the effects of a mHealth intervention (Health2Sync) versus standard insulin titration practices on usability, care process outcomes (including frequency of contacts with patients, time per interaction and average time for titration) and glycaemic control as measured by HbA1c.	The average total time taken to titrate patients was similar in both groups ($p>0.05$), however there were fewer failure of contacts reported with H2S ($p<0.01$) and time per interaction was also lower ($p<0.01$). Sensitivity analysis revealed that, excluding the influence of no contacts, H2S patients had a lower average time for titration ($p<0.01$). There was no difference in clinical outcomes as measured by HbA1c between groups ($p=0.75$).<br>A high acceptance and clinical utility of the H2S application was observed. Clinicians were happy to use H2S and found it easy and convenient for most patients. Importantly, this reduced frequency of contacts with patients, time per interaction and average time

						for titration (p<0.01). Patient selection for this communication intervention is important.
Brath et al. 2013 (44)	RCT blinded crossover	N=53 patients (30 female). Patients with increased cardiovascular risk treated for diabetes, high cholesterol and hypertension. Age group: 69.4±4.8 years old.	<u>mHealth technology/tool:</u> mHealth based remote Medication Adherence Management System (mAMS).<br><br><u>Tool purpose/ functioning</u>: to monitor and measure patients' adherence to medication and improve it with closed loop interactions. The system utilized Web and mobile apps as well as embedded microelectronic components to record the timestamp and amount of pills taken. mAMS elements are electronic medication blister, mobile phone based data gateway and remote telemonitoring service. Near Field Communication (NFC) enabled mobile phones to act as data gateway to transmit the blister data to the remote telemonitoring service, where it is seen by a physician.	52 weeks (4 weeks run-in with TAU; 20 weeks group 1 (or 2); 20 weeks group 2 (or 1); 4 weeks control phase; 4 weeks monitoring phase)	To study and analyze adherence to medication	Changes in adherence were significant for the diabetes medication only.<br>mHealth management is feasible and well accepted by patients with increased cardiovascular risk.<br>Electronic blisters can be used in a multi-medication regimen but need to be carefully designed for day-to-day application.
Doocy et al. 2017 (36)	Longitudinal cohort study (Pre-/Post and Follow up)	N=793 completed. Age group: >40 years old. Patients with hypertension and T2DM in primary health facilities in Lebanon that serve both Syrian refugees and Lebanese.	<u>mHealth technology/tool:</u> mHealth app.<br><br><u>Tool purpose/ functioning:</u> mHealth app included a personally controlled health record (PCHR), informational print-outs for patients on prescriptions, and lifestyle behaviors and served as an electronic medical record and decision support tool for providers. If patients move locations without their medical records, key diagnostic and treatment elements are available from the patient's cell phone subscriber identity module card, which constitutes the PCHR. The mHealth tool has the potential to improve quality and continuity of care, health literacy,	20 months	(1) To develop, adapt, and test existing standards and guidelines for treatment, including counseling, of persons with hypertension and T2DM (or both) and (2) to evaluate the effectiveness of a mHealth tool.	BP readings and blood sugar measurements significantly decreased following the implementation of treatment guidelines. BP readings also decreased after the mHealth phase.<br>There were a great proportion of patients for whom blood sugar, BP, weight, height, and BMI were recorded using the tablet compared with in paper records. However, only differences in BMI were significant (31.6% higher in app data as compared with

			mobility of medical records, and health outcomes for patients. Providers were trained in use of the app, and support was provided to health facilities for its implementation			paper records; p<.001). Data extracted from the mHealth app showed that a higher proportion of providers offered lifestyle counseling compared with the counselling reported in patients' paper records. There were statistically significant increases in all four measures of patient-provider interaction across study phases. Provider inquiry of medical history increased by 16.6% from baseline following guideline implementation and by 28.2% from baseline to mHealth implementation (p<.001).From baseline, patient report of provider inquiry regarding medication complications increased in the guidelines and mHealth phases by 12.9% and 59.6%, respectively, (p<.001). The proportion of patients reporting that providers asked other questions relevant to their illness increased from baseline through guidelines implementation by 27.8% and to mHealth implementation by 66.3% (p<.001). Follow-up scheduling increased from baseline to the guidelines phase by 20.6% and the mHealth phase by 39.8% (p<.001).
Dugas et al. 2018 (39)	Pre-post non-blinded RCT A parallel	N=27 (29; 2 drop-outs). T2DM patients Age group: ≥60 (67.56±5.81)	<u>Tool purpose/ functioning:</u> Investigators design and test a motivation psychology-based smart engagement system	13 weeks	(1) To explore the utility of the mHealth tool, the DiaSocial app, in	Between-groups results: age was unrelated to total adherence. There was a non-

	design with 5 conditions: TAU (n=5) vs. experimental condition: (T1) Patients using the app individually without clinician or peer engagement (n=5); (T2) Patients using the app with clinician engagement features (n=5); (T3) Patients using the app with peer engagement (n=6) (T4) Patients using the app with both clinician and peer engagement.	years old.	(MOSES), which is a SW application (The DiaSocial app) on a digital tablet device. The SW allows patients to record diabetes self-care activities (exercise, glucose, nutrition, and medication adherence), pursue goals, support communication between the patient and a health coach, support communication between peer patients, and visualize health status more easily by patients and providers.<br><br><u>mHealth technology/tool:</u> Samsung Galaxy Tab 3 preloaded with the DiaSocial app and a Fitbit One.<br><br><u>Instructions/Training:</u> Individuals were instructed to use the app daily, and were encouraged to earn points by recording their progress in achieving better diabetes self-care goals through managing glucose level, exercise, diet, and medication adherence. Participants who were assigned to teams met their teammates in person during the training session and were able to continue to interact online.		improving diabetes outcomes in a sample of older veterans.<br>(2) To explore whether individual differences in regulatory mode moderate the effectiveness of the mHealth intervention in increasing healthy behavior and improving clinical outcomes.<br>(3) Refine the personality attributes used to tailor the interaction with the application.	significant treatment effect (adherence and HbA1C change) and no differences in adherence levels across treatment.<br>Individual differences results: Assessment has a regulatory mode in improving adherence over time. Controlling for individual locomotion, treatment condition, and age, these results suggest that individuals higher in assessment might benefit from a self-monitoring oriented mHealth intervention to a greater extent than individuals low in assessment.<br>Adherence and healthy behaviors are related with locomotion: 1) Healthy behaviors increased among high locomotors for the first half of the study, but their motivation began to diminish as the study continued. Even so, high locomotors likely benefit from accumulating a greater number of points throughout the entirety of the study, 2) even as individuals high in locomotion were trending toward lower adherence scores in the latter half of the study, they were still marginally outperforming those low in locomotion.	
Holmen et al. 2014 (68)	3-arm prospective RCT: 2	N=151 (FTA=51; FTA-health counselling=50; Control	<u>Tool purpose/ functioning:</u> the mobile phone–based self-management system Few Touch Application (FTA) consisted	1 year	To test whether the use of a mobile phone–based self-management system	HbA level decreased in all groups, but did not differ between groups after 1 year.	✓

	intervention groups (one group received the mobile phone–based self-management system Few Touch Application; FTA and the other group received FTA and health counselling based on CBT and delivered by a diabetes specialist nurse for the first 4 months) and 1 control group.	group=50)<br>Follow–up available for 120 patients<br>T2DM patients Age group: FTA group: 58.6±11.8 years; FTA-HC group: 57.4±12.1 years old.	of a blood glucose–measuring system with automatic wireless data transfer, diet manual, physical activity registration, and management of personal goals, recorded and operated using a diabetes diary app on the mobile phone.<br><br><u>mHealth technology/tool:</u> The FTA system provided the user with a diabetes diary app designed to increase self-management through awareness, overview of relevant factors, and motivational feedback through symbols such as smiling faces and color codes in the app. The participants measured blood glucose level with a glucometer (LifeScan OneTouch Ultra Easy), which enabled automatic transfer of the measurement to the diary mobile app through a wireless Bluetooth connection and provided visual graphs, trend reports, and feedback through color coding (below normal, normal, and above normal). The app also consisted of a food habit registration system, a physical activity registration system, a personal goal-setting system, and a general information system. All info entered manually.<br><u>Instructions/Training:</u> Training was in person, a paper manual and a universal serial bus (USB) memory stick with further information were provided to participants. Technical support was available all weekdays.		with or without telephone health counseling could improve glycated hemoglobin A (HbA1C) level, self-management, and health-related quality of life compared with usual care.	Change in self-management skills (measured by the Health Education Impact Questionnaire; heiQ) and technique acquisition was significantly greater in the FTA-HC group after adjusting for age, gender, and education. Other secondary outcomes did not differ between groups after 1 year.<br>In the FTA group, 39% were substantial users of the app; 34% of the FTA-HC group were substantial users. Those aged ≥63 years used the app more than younger participants.
**Hunt et al. 2014 (64)**	**Two-group, crossover, repeated-measures**	**N=14**<br>**T2DM**<br>**Age groups: 6 participants between 19-50 years, 11 participants with ≥51 years old (n=17)**	**<u>mHealth technology/tool:</u> Diabetes Buddy® iPad app.**<br><br>**<u>Tool purpose/ functioning:</u> Diabetes Buddy® allows tracking of the recommended diabetes self-**	**3 months**	**To determine if the use of applications on Apple® iPad® will improve diabetes self-management and self-efficacy, as well as**	**Analysis of f-tests revealed no statistically significant change between-groups indicating the intervention did not impact self-efficacy in this sample. Some**

		Intervention (ipad) or control (journal) group	management behaviors of BGL monitoring, eating a healthy diet, exercising, and using medication. The app also allows emailing of data from within the application as well as unlimited logging of behaviors.<br><br><u>Instructions/Training:</u> The primary investigator distributed the Apple iPad devices and provided instructions on using the diabetes self-management application to log behaviors in a 1h meeting (grouped or individually according to participant preference). Participants also were instructed how to email logs to the primary investigator once per week using a single Gmail account established for the study. The control group participants were given instructions on how to log diabetes self-management activities in the provided journal. Contact information for the primary investigator was provided in case participants required assistance or had questions during the study period.		T2DM patients' clinical outcomes.	participants logged more self-management behaviors when they used the iPad and others logged more activities using the journal.
Kardas et al. 2016 (51)	Prospective parallel-arm RCT	N= 60<br>T2DM patients treated in the primary care settings in Lodz region, Poland Age group: 59.5±6.8 years old.	<u>Tool purpose/ functioning:</u> COMMODITY12 system, smartphone and wirelessly connected sensors.<br><br><u>mHealth technology/tool:</u><br>● a bluetooth-enabled glucometer, blood pressure reader and scale (Gluco-TelTM, Pressure-TelTM, and Weight-TelTM, respectively, BodyTel Europe GmbH, Bad Wildungen, Germany).<br>● a Bluetooth-enabled sensor of ECG, heart rhythm, and respiratory movements (BioHarnessTM, Zephyr	6 weeks	To assess system operability and trial feasibility, including patients' experience with their use of COMMODITY12 mHealth system. Secondary study aims included assessment of several metabolic parameters as well as patient adherence to the treatment.	All four layers of the COMMODITY12 system proved to work smoothly under real-life conditions, without major problems. All dimensions of experience with system use were assessed well, with maximum values for clearness of instructions, and ease of use. HRQoL as assessed with cumulative utility measures, improved significantly in COMMODITY12 system users. mHealth system

			**Technology, Annapolis, Maryland, USA).**<br>**● a triaxial accelerometer – already built in the SmartHub (mobile phone).**<br>**● MEMSTM (MeadWestvaco Corporation, Richmond, VA, USA) – a patient adherence monitor, which has been used to assess patient adherence with oral antidiabetic agents they were using (Metformin).**			**modestly improved glycaemic and BP control, assuring high level of patient adherence. Study proved that the COMODITY12 system is well accepted by T2DM patients taking part in the RCT, leading to several clinical benefits, and improved HRQoL. Nevertheless, before future commercialisation of the system, several minor problems identified during the study need to be addressed.**
Karhula et al. 2015 (52)	Two-armed RCT	N=471 (n=246 heart disease, n=225 T2DM patients finished trial).<br>Age heart group: 69.1±9.1 years<br>Age diabetes group: 66.2±8.6 years old.	mHealth technology/tool: patients monitoring through a mobile phone with specific software, a mobile personal health record (PHR) app, and a set of measurement devices connected to the patient's PHR account.<br><br>Tool purpose/ functioning: Patients measured their BP with a BP meter device, which was connectable to the mobile phone via Bluetooth, the value was automatically transferred to the PHR using a binary SMS. Other health parameters were body weight, BGL for diabetics, and step count for heart disease patients. Patients were instructed to measure and send these values manually via the mobile phone to the PHR once a week. Health coaches and patients were able to see the patients' measurements in the PHR and were advised to utilize them during health coaching phone calls. A self-management guide was given to the patients to increase their knowledge of their chronic disease.	1 year	Whether a structured mobile phone-based health coaching program, which was supported by a remote monitoring system, could be used to improve HRQL) and/or the clinical measures of T2DM and heart disease patients.	Withdrawal from the study was associated with the patients' unfamiliarity with mobile phones—of the 41 dropouts, 85% (11/13) of the heart disease patients and 88% (14/16) of the T2DM patients were familiar with mobile phones, whereas the corresponding percentages were 97.1% (231/238) and 98.6% (208/211), respectively, among the rest of the patients (p=.02 and p=.004). Withdrawal was also associated with heart disease patients' comorbidities—40% (8/20) of the dropouts had at least one comorbidity, whereas the corresponding percentage was 18.9% (47/249) among the rest of the patients (p=.02). The intervention showed no statistically significant benefits over the current practice with

			Remote Patient Monitoring System: intervention supported by the RPM system, eClinic, provided by Medixine Ltd (Espoo, Finland). The self-management server is the central component, providing services for the storing and accessing of RPM data (health parameters, personal care plan entered by the health coach, and data from the electronic health record (EHR). The HTTPS protocol was used for sending all data from the mobile app to the server.			regard to HRQoL—heart disease patients: beta=0.730 (p=.36) for the physical component score and beta=-0.608 (p=.62) for the mental component score; T2DM patients: beta=0.875 (*p*=.85) for the physical component score and beta=-0.770 (*p*=.52) for the mental component score. There was a significant difference in waist circumference in the T2DM group (beta=-1.711, *p*=.01). There were no differences in any other outcome variables. A health coaching program supported with telemonitoring did not improve heart disease patients' or T2DM patients' HRQoL or their clinical condition. Diabetes patients may be more prone to benefit from this kind of intervention
**Kim et al. 2014 (48)**	**Longitudinal cohort study, intervention and control group**	**N= 38 (3 dropouts) T2DM outpatients from Seoul St. Mary's Hospital. Age groups: Intervention group 51.8±10.3; control group 53.8± 9.0 years old.**	**<u>mHealth technology/tool:</u> Galaxy S-II Smartphone (Samsung Electronics, Suwon, Korea), CareSens-LINK blood glucose monitor (i-SENS, Wonju, Korea), and S(M)BPM-1 blood pressure monitor (Samsung Electronics), smartphone application Mobile Smartcare, version 1.0.7 (Samsung Electronics).**<br><br>**<u>Tool purpose/ functioning:</u> The blood glucose and blood pressure were self-measured by the patients and the recorded data were automatically transferred to the medical staff through the app Mobile Smartcare. The medical**	**3 months**	**To assess the efficacy of the smartphone-based health app for glucose control and patient satisfaction with the mobile network system.**	**Both the smartphone group and the control group showed a tendency towards a decrease in the HbA1c level after 3 months. In the more satisfied group (n=27), the HbA1c level decreased, in the less satisfied group (n=8), the HbA1c result increased showing values much worse than that of the no-smartphone control group.**

			**staff analyzed the data and sent recommendations and feedback tailored to the patient an average of once per week. If the BGL remained high, a warning message and recommendations for exercise and diet were sent to the user. If the user had hypoglycemia (<60 mg/dL) or did not record any BGL, the medical team called the user to change the insulin dose or recommend an early visit to the hospital.**			
Kim et al. 2016 (65)	Single-arm pilot study	N=29<br>T2DM patients<br>Age group: 53.9±9.1 years old.	<u>mHealth technology/tool:</u> Smartphone-based, diabetes care system (PSDCS) android-based app, mobile phone, Bluetooth glucometer (MyGlucoHealth; Entra Health Systems, Seoul, Korea), Bluetooth activity tracker (LG LifeGram LA11M-BS; LG Electronics, Seoul, Korea).<br><br><u>Tool purpose/ functioning:</u> The PSDCS was composed of 4 modules: 1) a glucose module, 2) a diet module, 3) a physical activity module, and 4) an SNS module. The glucose module consisted of a Bluetooth glucometer, feedback messages according to the glucose levels, recommendations for basal insulin dosage, and algorithms for detecting and coping with hypoglycemia and serious hyperglycemia. The diet module consisted of recording daily dietary intake and calculating total calories and nutrients based on a predefined food database. The physical activity module consisted of an activity tracker, a semi-automatic energy expenditure calculator, and video clips that guided resistance excise. The individualized target goal for diet and physical activity was set at baseline. The SNS module could motivate	12 weeks	To test the feasibility of HbA1c reduction with a patient-centered, smartphone-based, diabetes care system (PSDCS).	HbA1c and fasting plasma glucose levels decreased significantly from baseline. The frequency of glucose monitoring correlated with the magnitude of HbA1c reduction. The components of the diabetes self-care activities, including diet, exercise, and glucose monitoring, were significantly improved, particularly in the upper tertile of HbA1c reduction. There were no severe adverse events during the intervention.

			the participants by sharing their thoughts, opinions, and tips for diabetes self-care and displayed the scores and ranks according to the number of access to the PSDCS. The PSDCS also contained diabetes self-management educational material that provided detailed information concerning how to manage various diabetes-related conditions and specific situations, including sick-day rules. The website for physicians was developed to show the baseline characteristics, including anthropometric data, prescribed medications, and laboratory data, and to monitor readouts of the glucometer, amounts of daily calorie intake, and daily physical activity.<br><br>Instructions/Training: Face-to-face instruction session to understand the detailed use of the PSDCS.				
**Koot et al. 2019 (55)**	**Single-arm feasibility study**	**N=100 (primary outcome data available for 83 patients) T2DM patients with HbA1c levels of ≥7.5% from a single community health care facility in Singapore.<br>Age group: 53.5±9.6 years old.**	**mHealth technology/tool: GlycoLeap program a mobile lifestyle management program.<br><br>Tool purpose/ functioning: GlycoLeap program includes Glyco app, Accu-Chek performa (F. Hoffmann–La Roche Ltd) glucometer kit with lancets and test strips, a BodyTrace (BodyTrace Inc) wireless weighing scale, and a resistance band for strength training.**	**24 weeks**	**Assessing the potential effectiveness and feasibility of GlycoLeap as an add-on to standard care.**	**Program engagement started out high but decreased with time for all evaluated components. Self-reported survey data suggest that participants monitored their BGL on more days in the past week at follow-up compared to baseline and reported positive changes to their diet due to app engagement. Statistically significant improvements were observed for HbA1c with greater improvements for those who logged their weight more often. Participants also had a 2.3% reduction in baseline weight. User satisfaction was**	✓

						high with 74% and 79% of participants rating the app good or very good and claiming that they would probably or definitely recommend the app to others.
Larsen et al. 2010 (53)	Single-arm feasibility study	N=23 (one withdrawal) T2DM patients in a general practice setting in Oxfordshire<br>Age group: 57.6±12.0 years old.	<u>mHealth technology/tool:</u> Mobile phone (Nokia 6300) with pre-loaded SW (tþ Medical, Abingdon, UK), a blood glucose meter (OneTouch Ultra), and a Bluetooth cradle to link the meter to the phone.<br><br><u>Tool purpose/ functioning:</u> The mobile-phone app is an electronic diary with several questions embedded related to diabetes self-management behaviors and parameters, color-coded visual feedback was offered after each answer/parameter entered. A specialist diabetes nurse reviewed the patients' data every two or three days using a secure website, and contacted the patient if any of the following conditions were met: BGL were not received for three or more days, there were seven or more days with fasting blood glucose above 7.5 mmol/L, there was a single hypoglycaemic blood glucose reading below 4.0 mmol/L or any other situations which were of concern, at the nurse's discretion. The nurse provided general advice, motivation and assisted with technical problems. The nurse could also contact the general practice staff so that they could give appropriate medical advice and suggest any necessary dose modifications in addition to those already made by the patient.	6 months	To investigate the feasibility of a mobile-phone-based system that had recently commenced insulin treatment but remained poorly controlled.	Blood glucose control improved, as reflected by a mean decrease in HbA1c of 0.66% with the mean insulin dose increasing by 17 units. Blood glucose monitoring compliance was high, with readings available for 6.2 days per week, although the use of the mobile phone decreased during the study.<br>On average, the mobile phone diary was used for 3.5 days per week. Insulin dose adjustments were made throughout the study by all patients, but not as frequently as would be expected for the degree of hyperglycemia observed.

			Instructions/Training: Patients were instructed to measure their fasting blood glucose each day before breakfast and use these values to adjust their insulin dose using a self-titration algorithm, increasing the dose by 2 units every three days if two of the previous three days' fasting readings exceeded 6.7 mmol/L, provided that no reading was below 4.0 mmol/L.			
Li et al. 2020 (71)	Retrospective, propensity score-matched cohort study analysing longitudinal data from a clinical electronic health database.	N=2400 matched 1:1 T2DM patients Age group: 52.24±11.56 years old.	mHealth technology/tool: a mobile app, smart wearable medical devices (e.g., wireless BGL monitor, wireless BP monitor, pulse oximeter and body composition scale) a Web platform and a data sharing cloud platform. <br>Tool purpose/ functioning: Patients used the wireless glucose monitors and app to perform glucose checks at home. When they experienced hypoglycemia or hyperglycemia, the app provides tips to help them regulate their glucose levels. Patients also sent their results immediately to the support team about what to do. The care team and the service support team members would be notified about abnormal glucose levels. They then phoned the patient to inquire about their recent medication, diet, and exercise, and to help the patient in analyzing possible reasons. If necessary, they would invite the patient for further in-clinic consultation or guide the patient to adjust their diet or exercise by phone. Patients could also record their meals in the app and get feedback. According to an image or a description of the food, the team would provide an	12 months	To evaluate the effectiveness of a mobile-based intervention on glycaemic control in T2DM patients based on real-world population data.	This mobile-based intervention delivered by a multidisciplinary team to promote glycaemic control of T2DM patients led to increases in the control rates of HbA1c, FBG, and postprandial 2-hour blood glucose. These effects were best sustained within the first 6 months. Starting from the sixth month, intensive management might need to be conducted to maintain long-term effectiveness of this mobile-based intervention.

			**overall rating of the meal, comments on portion and nutrition, and suggestions on how to do better. Patients could log their exercise type and duration into the app. Patients had anytime access to educational information updated by the service support team.**			
Mora et al. 2017 (41)	6-month, prospective, multicenter, single-arm study	N=87 adults with type 1 and insulin-treated T2DM<br>Age group: 57.9±12 years old.	<u>mHealth technology/tool:</u> Accu-Chek Connect diabetes management system.<br><br><u>Tool purpose/ functioning:</u> The system consists of a BGL meter, smartphone application (app), and an online data management web portal. The meter connects wirelessly, by Bluetooth low energy technology, to the user's smartphone app, which provides multiple functions to facilitate diabetes management. The system provides automatic, wireless transfer of BG results from meter to the app and then to secure personal and clinical web portals. Users also have the option to share glucose data with others through text messages. A key feature of the system is the clinician portal home page, which automatically organizes the patient data, identifying patients who are at risk for acute glycaemic events, thereby providing clinicians the ability to triage patients according to greatest. Patients received timely guidance in therapy adjustments through remote (text or phone) and in person consults.	6 months	To assess the impact of using the Accu-Chek Connect diabetes management system on treatment satisfaction, diabetes distress and glycaemic control in adults with type 1 diabetes and insulin-treated T2DM.	Improvements in treatment satisfaction were observed at 6 months. Significant reductions in distress were also observed. A significant reduction in regimen-related distress was notable, from ''moderate distress'' to ''not distressed'' (DDS; Diabetes Distress Scale). Significant reductions in mean HbA1 and mean BG were also observed.
**Nes et al. 2012 (54)**	**Pre/post pilot study**	**N=15 (11 completed the study)<br>T2DM patients<br>Age: mean=59.6 years old.**	**<u>mHealth technology/tool:</u> The intervention included the use of smartphones (Samsung Omnia i 900) with four principal elements: (1) An Internet program that made possible the**	**3 months**	**To develop and test the feasibility of a three months web-based intervention, delivered by a smartphone to**	**The intervention was evaluated as supportive and meaningful. Most of the participants reported positive lifestyle changes. The**

	connection with a secure server to access the daily online entries; (2) Individualized written situational feedback; (3) Installed audio files with mindfulness and relaxation exercises; and (4) an installed healthcare tool the Few Touch Application (FTA).<br><br>Tool purpose/ functioning: The participants registered their eating behavior, medication taking, physical activities, and emotions three times daily using the mobile device (16-19 questions with predefined alternatives and comment fields). Participants received an SMS alert with a link to a secure website where the diaries could be filled in. They also registered their fasting blood glucose level in the morning diary. A therapist had immediate access to submitted diaries and used the situational information to formulate personalized feedback based on Acceptance Commitment Therapy (ACT). Participants also received a notebook with written exercises consisting of tasks to identify life values according to the ACT framework. In this notebook, they also could write about their current situation and their goals for health-related behavior.<br><br>Instructions/Training: An individual meeting between the participants and the researcher. Participants were taught how to use the smartphone, to fill in the diaries, to read the feedbacks, to hear the sound files, and to use the FTA. All the verbal information received was written down in an instruction manual, delivered together with the		support self-management in T2DM patients.	response rate to the daily registration entries was good and few technical problems were encountered.

			**smartphones, where the participants could consult if necessary. The participants could decide the most convenient time to receive the morning and evening diary. This agreement was concluded at the first meeting with the participants and was intended to adapt the intervention to the participants' daily routines. Also, the therapist, who had the main responsibility to write the feedback, contacted the participants by telephone before the beginning of the intervention (to create a relationship and get information about the need for support).**			
Orsama et al. 2013 (56)	RCT	N=48 (intervention group, n=24; control group, n=24) T2DM patients with high HbA1c or users of oral medication.<br>Age groups: intervention group 62.3±6.5; control group 61.5±9.1 years old.	mHealth technology/tool: mobile telephone, Monica SW application, personal health record Medinet, pedometer, glucometer.<br><br>Tool purpose/ functioning: After each upload, the app displays graphs reflecting the uploaded data about individual target values and information, motivation, and/or behavioral skills feedback message to support patient self-care. For each reported measurement, the patient received a feedback message based on the reported data and data reported earlier during the trial, based on a decision support algorithm. The objective was to encourage patients to initiate and maintain lifestyle changes appropriate to self-care of diabetes and hypertension by providing personalized, information, motivation-, and behavioral skills-rich feedback, based on patients' self-measured remote reported health parameters. For this purpose, the Information-Motivation-Behavioral Skills	10 months	To develop and evaluate a mobile telephone-based remote patient reporting and automated telephone feedback system, guided by health behavior change theory, aimed at improving self-management and health status in individuals with T2DM.	Intervention participants achieved, compared with controls and controlling for baseline, a significantly greater mean reduction in HbA1c and significantly greater weight reduction. Non-significant trends for greater intervention compared with control improvement in systolic and diastolic blood pressure were observed.

			Model was used as a basis for the formulation of feedback message contents. Intervention patients could also access their health record (Medinet) through a browser-based user interface offering the opportunity for patients to view their personal health information, including prescriptions, laboratory data, and their care plan. Study nurses scanned through the status of all intervention patients each week and contacted patients if warranted by their remote data reports.<br><br>Instructions/Training: The intervention group was instructed to carry out a BP measurement in the morning and evening of 1 day each week. They were also instructed to measure their weight once a week, in the morning. BGL was measured on 3 days each week. Selected intervention patients were instructed to take paired (pre-and postprandial) BGL measurements—before and 1–2 h after a meal—on blood glucose testing days. Patients were permitted to choose if and how they wanted to use the pedometer to measure activity. Patients were instructed to upload their health parameter data using the mobile app directly after making each measurement.			
**Prabhakaran et al. 2019 (45)**	**Prospective multicenter open-label cluster-RCT**	**N=3698 participants (3324 completed trial)<br>Multiple chronic conditions including diabetes, hypertension, current tobacco and alcohol use and depression<br>Age group: 55.1±11 years old.**	**mHealth technology/tool: The mWellcare system, an Android app built on the CommCare platform (Tablet).<br><br>Tool purpose/ functioning: The mWellcare system generates Electronic Decision Support (EDS) recommendations for the management of chronic conditions tailored to the**	**12 months**	**To evaluate the effectiveness of mWellcare, an mHealth system consisting of electronic health record storage and an electronic decision support for the integrated management**	**There was no evidence of difference between the 2 arms for systolic BP and HbA1c even after adjustment of several key variables for HbA1c and no differences in the changes between the 2 groups for tobacco and alcohol use or other**

			**participant's profile and risk level. It stored the health records electronically, enabling long-term monitoring and follow-up. It sends SMS reminders (to take medication and attend follow-up visits) to patients. The NCD nurse uses the mWell care system installed in a tablet to collect patient-specific clinical data. The mWell care system generates a Decision Support Recommendation (DSR) for the physician and recommends a treatment plan based on standard guidelines. Using the prompts of the DSR, the NCD nurse provides lifestyle advice to the patient. At every scheduled follow-up visit clinical parameters are recorded to generate a longitudinal trend/summary by the mWellcare system. Monthly reports on the number of participants reporting for the scheduled follow-up and the average change in clinical parameters.**<br><br>**<u>Instructions/Training:</u> Centralized training on the current clinical management guidelines to all physicians. Onsite training for orientation to the mWellcare system was conducted at each site. Training for NCD nurses in the management of hypertension, diabetes mellitus, depression, and tobacco and alcohol use and training on how to use the mWellcare system, supplemented by another 2 days of onsite supervision and support. In all mWellcare arm sites, simplified charts on the management of these conditions were displayed prominently.**		**of 5 chronic conditions (hypertension, diabetes mellitus, current tobacco and alcohol use, and depression) versus enhanced TAU among patients with hypertension and diabetes mellitus in India.**	**secondary outcomes. Authors did not find an incremental benefit of mWellcare over enhanced usual care in the management of the chronic conditions studied.**
Quinn et al.	Pilot pre- post	N=7	<u>mHealth technology/tool:</u> a mobile phone	4 weeks	To evaluate participant	Participants had high self-

2015 (62)	study	T2DM patients from community (PC)<br>Mean age=70.3 years old.	with a PCS (WellDoc, Inc., Baltimore, MD) SW and a web portal.<br><br><u>Tool purpose/ functioning:</u> The PCS is communication software that allows patients to enter diabetes self-care data into the phone (BG values, carbohydrate intake, medications, physical activity, and other diabetes management information). The PCS sends automated messages based on professional treatment guidelines to patients based on the entered BG data in addition to personalized messages sent by CDEs in the role of case manager, through an individualized patient Web portal. The PCS SW had available over 1,000 automated messages into a feedback algorithm provided for a given set of BG values or other clinical information entered by the participant. The algorithm displayed educational and motivational messages after the patient's self-reported data. The personalized Web portal also included a personal diabetes health record that patients were encouraged to update (laboratory values, eye examinations, foot screenings, results from provider visits), a learning library, and a historical log book.		self-efficacy and use of a mobile phone diabetes health intervention for older adults	efficacy and high readiness and confidence in their ability to monitor changes to control their diabetes. Participants demonstrated the ability to use the mobile intervention and communicate with diabetes educators.
**Quinn et al. 2008 (57)**	**Non-blinded Pilot RCT**	**N=30<br>T2DM<br>Age group: 51.04±11.03 years old.**	**<u>mHealth technology/tool:</u> Bluetooth® (Bluetooth SIG, Bellevue, WA)-enabled One Touch Ultra BG meter, adequate BG testing strips and lancets, and a Nokia (Espoo, Finland) 6682TM or 6680TM cell phone equipped with WellDoc's (WD'S) proprietary Diabetes Manager software.<br><br><u>Tool purpose/ functioning:</u> WD'S**	**3 months**	**To assess the impact on A1c of a cellphone-based diabetes management software system used with web-based data analytics and therapy optimization tools; to HCP adherence to prescribing guidelines and**	**Adults with type 2 diabetes using WellDoc's software achieved statistically significant improvements in A1c. The average decrease in A1c for intervention patients was 2.03%, compared to 0.68% for control patients. Of the intervention patients, 84% had medications titrated or**

	purpose is (1) to teach patients about dietary impacts on BG levels, (2) to direct patients to generate higher-quality BG data, and (3) to determine the effect of provided patient BG data, data analysis, and suggested therapy recommendations on Health Care Provider (HCP) prescribing behavior. Baseline patient data was entered into the HIPAA-compliant secured web-based registration system by the research team. Patient data were uploaded from the web server into the cell phone and integrated into the SW for personalized feedback. Once the BG value was received by phone, the WD's application on the cell phone was triggered. The software asked the patient to identify (label) the BG (e.g., "Before Breakfast?" "Bedtime?"). Once the BG was labeled, the patient was given feedback about the value related to the patient-specific target level and was shown his/her HCP-prescribed medication instructions. If the patient's BG levels were above or below his or her target levels, the patient was given real-time feedback on how to correct the BG level. The patient was then prompted to enter the medication dosage and carbohydrates eaten and sent to the WellDoc server. Patient data were analyzed by automated algorithms and by the research team. If there were no problems detected, the patient was given positive feedback. When a troubling BG value or pattern was detected, the patient either was directed to test or e-mailed several questions. Once the problem was identified, the patient was sent an e-mail with		adoption of technology.	changed by their HCP compared to controls (23%). HCP and patient satisfaction with the system was clinically and statistically significant. Intervention patients' HCPs reported the system facilitated treatment decisions, provided organized data, and reduced logbook review time.

			**educational material specific to that issue. WellDoc communicates suggested medication changes directly to patients and to the HCP that decides to implement or not the recommendations. Patient feedback was limited to nutrition, lifestyle stage of change, and self-management skills. If the Diabetes Team determined that the patient required a live session for nutrition or self-management help, the HCP was notified, and the patient was referred to a CDE and/or nutritionist within the patient's health care network. The HCPs in the intervention group received e-mails notifying the WDS patient's logbook and the report of patient data and trends was ready for review. Content for these reports was generated by the automated, proprietary statistical tools of the system and manual input from the WellDoc Diabetes team. In addition to patient BG data, the purpose of the reports was to alert the HCP about: (1) medication misuse; (2) patient dietary or self-management skill needs; (3) pharmacotherapy failures or adverse advents; and (4) medication titration recommendations to target A1c 6.5%.**			
Quinn et al. 2011 (60)	Cluster-RCT conducted in Primary Care practices in Maryland areas<br>4 arms study;<br>1) TAU (UC),<br>2) coach-only (CO), 3) coach	N=163<br>T2DM patients<br>Age: Mean=52.8 years old.	<u>mHealth technology/tool:</u> A mobile-phone, the WellDoc software, a web-based Diabetes ManagerTM System, and a One Touch Ultra 2 (LifeScan, Milpitas, CA) glucose meter and supplies.<br><br><u>Tool purpose/ functioning:</u> Patients received automated, real-time educational and behavioral messaging in response to individually analyzed blood	1 –year treatment period	To test whether adding the mobile app coaching and patient/provider web portals to community primary care compared with standard diabetes management would reduce HbA1c levels in T2DM patients.<br>To report changes in	The mean declines in HbA1c were 1.9% in the maximal treatment group (CPDS) and 0.7% in the TAU group, a difference of 1.2% over 12 months.<br>Appreciable differences were not observed between groups for patient-reported diabetes distress, depression, diabetes

	PCP portal (CPP), and 4) coach PCP portal with decision-support (CPDS).		glucose values, diabetes medications, and lifestyle behaviors communicated by mobile phone. Providers received quarterly reports summarizing patient’s glycemic control, diabetes medication management, lifestyle behaviors, and evidence-based treatment options.		patient-reported diabetes symptoms, diabetes distress, depression, and other clinical (blood pressure) and laboratory (lipid) values.	symptoms, lipid or BGL. The combination of behavioral mobile coaching with blood glucose data, lifestyle behaviors and patient self-management data individually analyzed and presented with evidence-based guidelines to providers substantially reduced glycated hemoglobin levels over 1 year.
Quinn et al. 2016 (72)	Cluster RCT	N=118<br>T2DM patients from Primary care.<br>Stratified into two age groups: <55 & ≥55 years old.	<u>mHealth technology/tool:</u> Intervention patients received a patient coaching system (PCS) and provider clinical decision support (Quinn et al., 2011). The PCS included a mobile diabetes management software application (MDMA) on a mobile phone. All patients in both study groups received a One Touch Ultra 2TM (LifeScan, Milpitas, California) glucose meter and glucose testing supplies.<br><br><u>Tool purpose/ functioning:</u> The PCS included a mobile diabetes management software application (MDMA), which allowed patients to enter diabetes self-care data (blood glucose values, carbohydrate intake, medications, and other diabetes management information) and receive automated, real-time messages that were educational, behavioral, motivational, and specific to the entered data. Each automated message was one of 1,000 such messages incorporated into a feedback algorithm. The patient-entered diabetes self-care data were intermittently reviewed by “virtual” case managers called diabetes educators, with whom patients could	1-year treatment period	To test whether a mobile phone coaching system and patient/provider web portals, compared with standard diabetes management, would result in a greater reduction in HbA1c levels in T2DM patients.	The intervention resulted in greater 12-month declines in HbA1c, compared with usual care, for patients in both age groups. Among older patients, HbA1c changed by −1.8% in the intervention group and −0.3% in the control group. Among younger patients, HbA1c changed by −2.0% in the intervention group and −1.0% in the control group. The mobile health intervention was as effective at managing Type 2 diabetes in older adults as younger persons.

speak on the telephone but were encouraged to communicate electronically. The diabetes educators could supplement MDMA messages with messages based on longitudinal diabetes self-care data trends. These supplemental messages were sent electronically to a patient web portal. The patient web portal, like the MDMA, was part of the PCS. It consisted of a secure messaging center for patient-provider communication; a personal health record with additional diabetes information, such as laboratory values, eye examinations, and foot screenings; a learning library; and a logbook to review historical data. In addition to receiving access to the patient web portal, each patient in the intervention group received a mobile phone, a 1-year unlimited data and service plan, and study PCS SW. Each patient in this group also received an electronic diabetes self-care action plan every 2.5 months to support improved diabetes self-care and to serve as pre-visit summaries for PCP visits. Providers assigned to the intervention group could access analyzed patient diabetes self-care data, linked to standards of care and evidence-based guidelines, via a provider web portal. They were trained on how to access the provider web portal on office personal computers (PCs) and were informed that their patients received a PCS. Besides, they received quarterly reports (more often if needed) that summarized patients' glycemic and metabolic control, adherence to medication, self-management skills, and relevant evidence-based guidelines.

Quinn et al. 2014 (61)	Cluster RCT (secondary analyses from the study Quinn et al. 2011)	N=117 (n=55 TAU; n=62 intervention group) Primary Care T2DM patients Age: Control group=53.3±8.4; Intervention group=52.0±8	<u>mHealth technology/tool:</u> Intervention patients received a patient coaching system (PCS) and provider clinical decision support (Quinn et al., 2011). The PCS included a mobile diabetes management software application (MDMA) on a mobile phone. All patients received a One Touch Ultra 2TM (LifeScan, Milpitas, California) glucose meter and glucose testing supplies.<br><br><u>Tool purpose/ functioning:</u> The coaching system involved patients using mobile phones to record information about their diabetes self-management. Patients received real-time (automatic) and personalized coaching feedback consistent with their treatment plans. Physicians could review patient-recorded data accessible through the provider Internet portal and received quarterly facsimile reports that included diabetes treatment recommendations.	1 year	To determine how a mobile-phone-based coaching system for diabetes management influences physician prescribing behavior.	A higher percentage of patients in the intervention group had modification and intensification of incretin mimetics during the 1-year study period (9.7% vs 0.0% and 8.1% vs 0.0%). A higher percentage of patients in the intervention group had modification and intensification of metformin (24.2% vs 7.3%). The overall difference in physician prescribing of oral antihyperglycemic medications was not statistically significant. Differences in physician prescribing behavior were modest, and do not appear to be large enough to explain a 1.2% decrease in HbA1c.
**Sittig et al. 2020 (50)**	**Pilot pre/post trial**	**N=20 T2DM patients Age group: 54.7±10.4**	**<u>mHealth technology/tool:</u> CapABILITY mHealth app.**<br><br>**<u>Tool purpose/ functioning:</u> CapABILITY and its affiliated trigger (text) messages integrate components from social-cognitive theory (SCT), Fogg Behavior Model (FBM), and persuasive technology into the interactive health communications framework. Participants interacted with the capABILITY app and received (or not received) text messages in alternative blocks. The capABILITY app alone is the control condition along with trigger messages including spark and facilitator messages. CapABILITY covers 3 main**	**9 weeks**	**(1) To determine the impact of a mHealth app that incorporated theory-driven trigger messages following the FBM and targeted self-efficacy, knowledge, and self-care. (2) To assess the feasibility of cap ABILITY app in modifying these behaviors.**	**Pre-post-intervention results indicated statistical significance on 3 of the 7 health survey measures (general diet, exercise and BGL). When only analyzing the high and mid users of capABILITY, authors found a statistically significant difference in both self-efficacy and exercise. Although the ANOVA did not reveal any statistically significant differences across groups, there is a trend among spark conditions to respond more quickly**

			**modules (3 weeks in each module): diet, exercise, and self-management (e.g., medication adherence and glucose monitoring). Within each module, new material is delivered each week. The majority of the educational content is retrieved from the ADA, provided through media and text. The media files consisted of short (2-3 min) videos of one of the researchers highlighting key educational content areas such as strategies for carbohydrate counting and providing weekly content overview videos (it can be paused, rewound and fast-forwarded). The text files have hyperlinks in case the participants want to read the complete documents (e.g. healthy recipes).**<br><br>Instructions/Training: **The design of capABILITY app is based on clinical experts and T2DM patients (who did not participate in the study) focus group meetings. Participants attended a launch event where they consented, were provided education on how to use capABILITY, and had capABILITY downloaded on their device.**			**following the receipt of the message.**
Steinert et al. 2016 (66)	Pre-post trial	N=36.<br>Age group: >60 years old (69,6±5,8)<br>T2DM patients	mHealth technology/tool: Smartphone and My Therapy diabetes management app.<br><br>Tool purpose/ functioning: My Therapy app allows the user to transfer selected health-related goals to a simple daily to-do list. The app acts as a reminder of taking medication (type, time of administration, dosage), measurements (e.g., blood glucose concentration, body weight) or physical activities (e.g., go for	3 months	Examine usage and benefits of a smartphone app for self-monitoring	Patients use smartphone app intensely (90 % on a daily basis).<br>Significant improvements in medication adherence and psychological well-being were revealed.<br>Motivational elements did not influence the usage behaviour.

			a walk, physiotherapy). Patients can scan barcodes of medication and enter BGL and other measurements, automatically stored in the app.			
Sun et al. 2019 (67)	Two-armed RCT	N=91 (Intervention group n=44; Control group n=47) Age group: >65 years old T2DM Patients	mHealth technology/tool: mHealth management app (app-based dietary software), pedometer, glucometer and medical server.<br><br>Tool purpose/ functioning: Patients upload (via Bluetooth) the glucometer data to the mHealth management app, which is then automatically transmitted to a medical server. The medical teams logged on to the system and sent medical advice and reminders to patients to monitor their BGL via the personal messaging app or telephonically every 2 weeks. Patients in the control group received a free glucometer and were followed up through conventional outpatient clinic appointments. For the control group patients, no limitations were imposed to the number of visits; however, they were instructed to monitor and record their BGL regularly.<br>The study dietitian offered guidance for BGL monitoring and provided dietary advice based on the individual BGL via the mHealth app based on daily dietary intake that patients upload to the app-based diet management SW.<br>Information pertaining to physical activity (daily calorie expenditure) is obtained by text message. Patients' text pedometer data to the study personnel.<br>Patients are provided with guidance related to aerobic and resistance-based exercises.	6-month intervention (Including follow-up after 3 and 6 months)	To investigate efficacy and safety of mobile phone–based telemedicine apps for management of older Chinese T2DM patients.	After 3 months, patients in the intervention group showed significant improvement in postprandial plasma glucose level.<br>After 6 months, patients in the intervention group exhibited a decreasing trend in postprandial plasma glucose and HbA1c levels compared with the baseline and those in the control group.

			<u>Instructions/Training:</u> Patients are trained but no more specific information is mentioned in the study.			
**Takenga et al. 2014 (43)**	**RCT**	**N=40<br>T2DM patients in the Democratic Republic of Congo (Goma and Butembo)<br>Age: intervention group=53.3±10.7; control group=53.35±9.59 years old.**	**<u>mHealth technology/tool:</u> Mobil Diab system including Mobil Diab app, web-based portals (patient, doctor, hospital administrators, and system administrator) integrated to the system and hosted from the central platform, a smartphone, glucometer.<br><br><u>Tool purpose/ functioning:</u> Mobil Diab app: Data is automatically synchronized in the background with the online system so that medical care providers receive these data in real-time. To record diabetes-related data, patients are presented with intuitive screens and they can enter data. These data may include, amongst others, the blood glucose measurement, insulin intake, sport done with duration, blood pressure measurements, and body weight and size. Data acquisition can also take place automatically from glucose meters. Data synchronization is done automatically and in two directions. Therapy plans, instructions, and recommendations sent by the doctor from the doctor portal are received directly in the mobile app. For patients without smartphones, can still get these doctors' feedbacks in the protected patients' portal, through their email address, or as SMS if this service was activated.<br>*Web-Based Applications of Mobil Diab*: Clinicians have a structured and understandable view of patients' data to make appropriate decisions on therapy**	**2 months**	**Validation of Mobil Diab System in the African context**	**The mean HbA1c for the intervention group was 8.67% before the start of the trial and could be reduced to 6.89% at the end. This value of 1.78% in the improvement of HbA1c indicates that the risk of diabetes complications has been reduced or postponed, thus leading to cost savings. The mean BG standard deviation was 48.6 mg/mL for the control group whereas; this could be reduced to 33 mg/dL for the intervention group at the end of the trial.<br><br>Patients found the system as a helpful tool since it helped them increase their motivation for regular HbA1c control, motivated them to fix some goals or targets values for BGL control for the future, and helped them to control their meal since they could access easily the database containing the amount of carbohydrate for each meal and the portion they were taking. Medical care personnel listed such benefits as follows: the system allowed them to coach several patients at the same time and at distance. Some**

			adjustment. Doctors can receive messages from their patients directly in this portal. For emergency cases, an SMS is automatically generated and sent to the treating physician, to alert him to check the critical situation in the portal and be able to give direct instructions to the patient.<br>*The architecture of the Telemedical Platform:* The platform helps consumers track health, wellness, and vital information using a highly secure infrastructure. It allows consumers to share information with their health care professionals and family. Mobility is guaranteed by integrating mobile apps and web-based applications. Moreover, interfaces to hospital information systems and practice management SW are supported.			disadvantages such as costs for internet connection and time needed for getting used to the app were preoccupations of both categories of the users. Improvements highlighted for both categories of users were: (i) Extend the drug list and meal database in the app to include all local foods, and applied drugs, since some of them were missing, (ii) More sensitizing activities and training sessions about the system, (iii) Include the support of the glucose-measuring device kit in the model, (iv) Create a framework for sports activities for patients.	
Torbjornsen et al. 2014 (69)	3-armed RCT	N=124<br>T2DM patients<br>Age group: FTA (n=51) 58.6±11.8 years old; FTA with health counselling (n=50) 57.4±12.1 years old; control group (n=50) 55.9±12.2 years old.	mHealth technology/tool: the Few Touch Application (FTA) diabetes diary with or without health counseling<br><br>Tool purpose/ functioning: a diabetes diary. The smartphone provided was a HTC HD Mini based on the Windows Mobile 6.5 operating system, and the blood glucose meter was the OneTouch Ultra Easy from LifeScan. The phone and the blood glucose meter were linked using Bluetooth wireless communication so that glucose measurements were automatically transferred to the diabetes diary part of the FTA on the phone.<br>The FTA is a self-management tool that comprises 5 main elements accessible to the user: (1) the blood glucose data	1 year intervention, with 2 follow-up assessments at 4 and 12 months.	To evaluate whether the introduction of technology-supported self-management using the FTA diabetes diary with or without health counseling improved HbA1c levels, self-management, behavioral change, and health-related quality of life, and to describe the sociodemographic, clinical, and lifestyle characteristics of the participants.	No differences in HbA1c between groups after 4 months, but a decline in all groups. Changes in self-management measured using the health service navigation item in the Health Education Impact Questionnaire (heiQ), with significant improvements in the FTA group compared to the control group and in the FTA with health counseling group compared with both other groups. Authors maintain it would indicate an improvement in the ability of patients to communicate health needs to their health	✓

			management system, (2) food habits data management system, (3) physical activity data management system, (4) personal goal-setting system, and (5) general diabetes information look-up system.<br>The BGL were transferred directly from the blood glucose monitoring system to the app via Bluetooth. The diet and physical activity systems enabled an easy way of entering such data manually into the diabetes diary by the user.<br><br>Instructions/Training: Both the FTA group and the FTA with health counseling group are trained to use the mobile phone-based system at the start-up meetings, which included a demonstration of the diabetes diary. They are also provided with a manual that contains instructions on the use of the smartphone, whereas the instructions for the FTA were supplied in the form of a paper-based handbook and on a universal serial bus (USB) memory stick. In addition, the consent form informed the participants about the diary and its specific procedures. A telephone support service was available to answer questions and to help the participants with technical aspects during weekdays from 9:00 to 15:00. The participants in the FTA with health counseling group were given additional training about how to send and receive secure messages to the diabetes nurse.			care providers. Furthermore, the FTA group reported higher scores for skill and technique acquisition at relieving symptoms compared to the control group. There were no significant changes in any of the domains of the SF-36.
Turner et al. 2009 (58)	Pre-post exploratory trial	N=23<br>T2DM patients<br>Age group: 58±12 years old.	mHealth technology/tool: a mobile phone with a preloaded SW application, and a blood glucose meter linked to the phone via a BluetoothTM cradle.	3 months	To assess the feasibility and acceptability of telehealth monitoring and support for insulin	The decrease in HbA1c at 3 months was 0.52% (0.91) with an insulin dose increase of 9 units. A mean (SD) of 160 (93)

			**Tool purpose/ functioning: SW used in the study was based on a commercially available system t+ Diabetes (supplied by t+ Medical, Abingdon, UK). It provides real-time data transmission and feedback to patients on their mobile phone through (i) transmission of blood glucose test results and real-time feedback of trends to the mobile phone; (ii) an electronic patient diary with the facility to record insulin doses; and (iii) a facility to transmit blood pressure results and weight. Patients were provided with immediate feedback including summaries and charts of entered data allowing them to monitor and make self-management decisions. Nurse and clinicians have access to web-based summary and feedback screens and the capacity for automated messaging to patients' mobile phones for alerts and reminders.**<br><br>**Instructions/Training: 30 to 60 minutes of telehealth training was given by the nurse at the general's practice first visit. A short training and support manual containing information about the use of the system and viewing data was given to each patient. Following the initial training, session patients were provided with a telephone number for the telehealth nurse and were asked to contact her if they had any questions.**		**initiation and adjustment.**	**blood glucose reading was transmitted per patient in these 3 months. Practice nurses and general practitioners viewed the technology as having the potential to improve patient care. Most patients were able to use the equipment with training and welcomed the review of their BGL by a telehealth nurse.**
Waki et al. 2014 (49)	Non Blinded RCT	N=54 (intervention group: n=27; control group: n=27). T2DM patients (diagnosed >5 years ago) Age: Intervention	mHealth technology/tool: Smartphone (NEC, Tokyo, Japan: MEDIAS WP N-06C), NFC-enabled glucometer (Terumo, Tokyo, Japan: MS-FR201B) and Bluetooth-enabled BP monitor (Omron,	3 months	To develop and test the feasibility and effectiveness of DialBetics, a real-time, partially automated	HbA1c and fasting blood sugar (FBS) values declined significantly in the DialBetics group: HbA1c decreased an average of 0.4% (from 7.1±1.0%

	group=57.1±10.2 years old; control group=57.4±9.4 years old.	Kyoto, Japan: HEM-7081-IT), pedometer (Omron HJ-720IT) with the adapter (Omron HHX-IT1), and scale (Omron HBF-206IT), all devices paired with a unique communicator that transmitted the readings by wireless network to the DialBetics server.<br><br><u>Tool purpose/ functioning:</u> DialBetics is composed of 4 modules: 1) data transmission, 2) evaluation, 3) communication and 4) dietary. Data transmission module: patients' data—blood glucose, blood pressure, body weight, and pedometer counts—are measured at home and sent to the server twice a day right after the patients' measurement, the first 3 upon waking in the morning, then blood glucose, blood pressure, and pedometer readings at bedtime. In the evaluation module, data are automatically evaluated following the Japan Diabetes Society (JDS) guideline's targeted values—optimally, blood glucose below 110 mg/dl before breakfast, below 140 mg/dl at bedtime; blood pressure below 130/80 mmHg; and pedometer count above 10,000. Readings defined as abnormal—blood glucose above 400 mg/dl or below 40 mg/dl, and systolic blood pressure above 220 mmHg—are reported to a physician that will check the data and interact with the patient if necessary. Communication module: (a) the patient's voice/text messages about meals—main dish of a meal—and exercise that is not counted by a pedometer—the type of exercise and its duration—are sent to the server; (b) message processing, if by voice input, is converted to text and matched with text		interactive system to interpret patients' data - biological information, exercise, and dietary content calculated from a message sent by patients-, and respond with appropriate actionable findings, helping the patients achieve self-management.	to 6.7±0.7%) compared with an average increase of 0.1% in the non-DialBetics group (from 7.0±0.9% to 7.1±1.1%); The DialBetics group FBS decreased an average of 5.5 mg/dl compared with a non-DialBetics group average increase of 16.9 mg/dl. BMI improvement—although not statistically significant because of the small sample size—was greater in the DialBetics group. DialBetics was shown to be a feasible and effective tool for improving HbA1c by providing patients with real-time support based on their measurements and inputs.

			in the DialBetics database; (c) advice on lifestyle modification, matched to the patient’s input about food and exercise, is sent back to each patient immediately after the patient’s input. The system can search for food and exercise input by patients among 3000 foods and 20 exercises in the database. The success rate for matching was 81.6%.<br><br>Predetermined thresholds and safety parameters for blood glucose and blood pressure were programmed in the database with readings outside threshold limits triggering an email automatically sent to the principal investigator and the diabetes nurse. The specialists provided technical troubleshooting and monitoring for devices, data portal, database, and alerts. The database triggered alerts for missed or late readings, the alerts sent to the nurse and the participants, with the nurse emailing the participants (after 1 week missed) or phoning (with 2 weeks missed), encouraging them to measure their data, and involving the specialists and experts if their help was required. The participants contacted the nurse by smartphone or email only for equipment failures, technical questions, or in response to alerts. For questions related to their health status, they were asked to consult their primary physicians.<br><br><u>Instructions/Training:</u> The research team trained each participant to take measurements and transmit them properly, and to understand the readings.				
Wayne & Ritvo, 2014	Single-arm pilot study	N=21 (19 completed trial) T2DM ethnically diverse	<u>mHealth technology/tool:</u> The Connected Health and Wellness platform (CHWP)	24 weeks	To develop and test a smartphone-assisted	12 subjects had baseline HbA1c levels >7.0% and a	

(37)		lower SES from the Black Creek Community Health Centre (Toronto) with a minimal prior smartphone experience Age group: 55.6±12.3 years old	Health Coach app 1.0 version (NexJ Systems Inc) on a Blackberry Curve 8900.<br><br>Tool purpose/ functioning: The coach app is designed to support multi-channel communications between clients and health coaches and supportive family members. The app electronically tracks health behaviors (e.g., exercise, diet, stress reduction practices) and self-monitors health data (e.g., blood glucose, blood pressure, mood, pain, energy). Wellness plans were collaboratively created in multiple interactions focused on exercise instruction and reviews of electronic monitoring entries, with diet and medication guidelines set by primary care physicians and dieticians.		intervention to improve behavioral management of T2DM in lower SES population	mean reduction of 0.43% (SD=0.63) (p<.05) with minimal changes in medication after the intervention.	
Wayne et al. 2015 (38)	**Pragmatic RCT**	**N=131 (Intervention group n=67, control group n=64) T2DM lower-socioeconomic status patients Age group: 53.2±11.3 years old**	**mHealth technology/tool: Samsung Galaxy Ace II mobile phone running Google Android Ice Cream Sandwich (Android 4.0.2) for the study intervention period, with a data-only carrier plan.**<br><br>**Tool purpose/ functioning: Connected Wellness Platform (CWP) provided by NexJ Systems, Inc. Which supported participants in health-related goal setting and progress monitoring. Participants could track key metrics, notably blood glucose levels, exercise frequency/duration/intensity, food intake (via photo journaling) and mood. They could communicate with their health coach at any time in the 24-hour cycle via secure messaging, scheduled phone contact, and/or during in-person meetings. The mean total contact (for all these activities) was 38 minutes/week**	**6-month intervention (follow up at 3 and 6 months)**	**To evaluate a health coach intervention with and without the use of mobile phones to support health behaviour change**	**While both groups reduced their HbA1c levels, there were no significant between-group differences in change of HbA1c at 6 months using intention-to-treat or per-protocol principles. However, the intervention group did achieve an accelerated HbA1c reduction, leading to a significant between-group difference at 3 months. This difference was reduced at the 6-month follow-up as the control group continued to improve, achieving a reduction of 0.81% compared with a reduction of 0.84% in the intervention group. Intervention group participants also had**	✓

(SD 25). All health data entered by participants into the CWP were immediately visible to health coaches through a secure, Web-accessible portal. Although participants were encouraged to use the system daily, individual usage patterns varied. Participant data and SW-enabled communication required two-way, certificate-based authentication and passwords that were stored in encrypted columns. The CWP exceeds Canadian privacy standards for software carrying health information. Based on patient goals, HCs used the 24-hour/day logging function to guide healthy lifestyle choices, while providing support when clients diverged from intended health goals.

<u>Instructions/Training</u>: Health Coaches with expertise in behaviour-change counselling and chronic disease management guide participants of both groups in planning and reaching health targets aimed at reducing HbA1c. All attended weekly seminars prior to and throughout the trial where they received training and participated in weekly team meetings where they discussed applications of behaviour theory in specific strategies for each participant. During trial, both intervention and control group participants assisted in an exercise education program (exercise classes, resistance training with weights and bands, and cardiovascular exercise using a treadmill and stationary bicycles) that featured exercise prescription, monitoring and adherence support. Participants were monitored on both an individual and group basis by

significant decreases in weight and waist circumference while controls did not.
Both groups reported improvements in mood, satisfaction with life, and HRQoL.

			trainers during exercise sessions and patients with T2DM were provided with special BG testing before and after sessions.			
Yoo et al. 2009 (46)	RCT	N=123 (control group: n=54; intervention group: n=57) Overweight T2DM and hypertension patients from a university hospital (Korea University) and from a community healthcare centre (Guro-Gu Public Health Centre) Age: Control group=59.4±8.4 years old; Intervention group=57±9.1 years old.	mHealth technology/tool: The Ubiquitous Chronic Disease Care (UCDC) system is composed of a patient-based cellular phone and web-based physician communication. is com. Patients in the UCDC group received a cellular phone (LG-SV280; LG Electronics, Seoul, Korea) with a modular blood glucose measuring device (Anycheck; Insung Information Co., Seoul, Korea), strips, and lancets. Also an automatic blood pressure monitoring device (T5M; Omron, Kyoto, Japan), as well as body weight scales (HD308; Tanita, Tokyo, Japan). <br>Tool purpose/ functioning: First, the UCDC system sent out an alarm on the cellular phone to remind the participant to measure their blood glucose, blood pressure twice a day (before breakfast and bedtime) and body weight once a day (before breakfast). The Anycheck device attached to their cellular phone conducted the glucose measurements and automatically sent the results to a central study database. As soon as participants transmitted their glucose measurement through their cellular phones, they immediately received messages of encouragement, reminders, and recommendations. Second, the UCDC system automatically recorded participant's exercise time using the SMS which was predefined according to each patient's daily schedule.	3 months	To investigate the effectiveness and applicability of the Ubiquitous Chronic Disease Care (UCDC) system using cellular phones and the internet for overweight patients with both Type 2 diabetes and hypertension	After 12 weeks, there were significant improvements in HbA1c in the intervention group compared with the control group. A significant reduction in systolic and diastolic blood pressure was observed, as well as improvements in total cholesterol, low-density lipoproteincholesterol and triglyceride levels in the intervention group. There was a significant increase in adiponectin levels in the intervention group compared with the control group, although high-sensitivity C-reactive protein and interleukin-6 levels did not change in either group.

			Participants replied via an automatic answer system whether or not they exercised. Third, participants received information via SMS three times a day regarding healthy diet and exercise methods, along with general information about diabetes, hypertension, and obesity. Furthermore, using the internet website, physicians could follow participant's trends in blood glucose levels, blood pressure and body weight changes, allowing them to send individualized recommendations.			
Young et al. 2020 (63)	**2-armed RCT**	**N=287 (control group n=155; intervention group n=132). T2DM patients Age group: 59.07±11.4**	**<u>mHealth technology/tool:</u> A wearable tracking device (initially, Basis Peak, then Garmin VivoSmart Heart Rate [HR]), an iPod Touch, the MyFitnessPal mobile app, and 2 connectors Apple HealthKit and MyChart to accomplish the automatic transmission of data to the an Electronic Health Record (EHR).<br><br><u>Tool purpose/ functioning:</u> Intervention entailed nurse health coaching and mHealth technology to track patient-generated health data (PGHD) and integrate these data into an EHR. The tracking device generated real-time information about steps taken, distance walked, active minutes, heart rate, and hours of sleep at night and synced the data to either an iPhone operating system mobile phone and/or iPod Touch. Participants can log and track nutritional consumption in the mobile app. In-person or telephonic technical support is available for all participants throughout the duration of the study. PGHD were integrated into the EHR when participants synchronized the**	**9-month intervention (follow-up measures at 3 and 9 months)**	**To evaluate the effectiveness of a nurse coaching program using MI paired with mHealth technology on diabetes self-efficacy and self-management for persons with T2DM.**	**The participants in the intervention group had significant improvements in diabetes self-efficacy management behaviors (measured with Diabetes Empowerment Scale) and a decrease in depressive symptoms compared with usual care at 3 months (measured with Patient Health Questionnaire-9), with no differences in the other outcomes. The differences in self-efficacy and depression scores between the 2 arms at 9 months were not sustained. The participants in the intervention group demonstrated a significant increase in physical activity (steps per week) at 3 and at 9 months.**

			**activity tracker to their device. Participants, primary care providers, and nurse health coaches could view trends in activity levels, sleep, and nutritional intake on either their smart device or on a computer.**			
Yu et al. 2020 (35)	Pre-Post longitudinal study	N=97<br>T2DM patients from an outpatient in Hualien (Taiwan).<br>Age group: 65.29±7.08 years old.	mHealt technology/ tool: The Intergenerational Mobile Technology Opportunities Program (IMTOP) including tablet and a diabetes self-management app (IMTOP app) and a web-based database.<br><br>Tool purpose/ functioning: The IMTOP is an mHealth intervention that recruits college-student tutors to help older patients to learn how to use mobile technology and a health tracking app for diabetes self-management.<br><br>Instructions/Training: The IMTOP intervention comprised 8 small-group training weekly sessions (10-12 people per class) followed by a 4-week technical support period. Training themes included tablet use skills, an introduction to the Internet and the online community, use of mobile applications to fulfil daily and health care needs, peer and professional support for diabetes care, and diabetes self-management knowledge and skills. In each class, a lecture was delivered by a trained IMTOP research staff member. College student tutors, who were trained by attending online courses, conducted simultaneous digital technology skill sessions and provided individualized support to participants. College student tutors either volunteered to or received college service learning credits as a result.	8-month intervention with follow-up measures at 4 and 8 months	To test the longitudinal efficacy of a mHealth intervention for older T2DM patients in rural Taiwan.	At 4 months, improvements in self-care behaviors were reported in diet, exercise, smoking, and BGL. Patients paid less endocrinology clinic visits, spent less on endocrinology medications, and improvements in fasting blood glucose and total cholesterol were observed. At 8 months, the statistical significance of improvements in diet and smoking were maintained, and the averaged endocrinology clinic visits remained less than baseline. However, more frequent occurrence of diabetes symptoms were reported at both follow-ups.

			The 4-week technical support was provided by research staff members as an additional help. During and continually after the intervention period, participants were invited to use on their tablets a diabetes self-management app (IMTOP app) previously developed by the research team, in which they could record their diet, physical activity, foot check, water consumption, medication adherence, BGL, BP, weight, and mood. Recorded data were transmitted via the Internet to the web-based database.			
Yoo et al. 2009 (46)	**RCT**	**N=123 (control group: n=54; intervention group: n=57) Overweight T2DM and hypertension patients from a university hospital (Korea University) and from a community healthcare centre (Guro-Gu Public Health Centre) Age: Control group=59.4±8.4 years old; Intervention group=57±9.1 years old.**	**<u>mHealth technology/tool:</u> The Ubiquitous Chronic Disease Care (UCDC) system is composed of a patient-based cellular phone and web-based physician communication. is com. Patients in the UCDC group received a cellular phone (LG-SV280; LG Electronics, Seoul, Korea) with a modular blood glucose measuring device (Anycheck; Insung Information Co., Seoul, Korea), strips, and lancets. Also an automatic blood pressure monitoring device (T5M; Omron, Kyoto, Japan), as well as body weight scales (HD308; Tanita, Tokyo, Japan).<br><br><u>Tool purpose/ functioning:</u> First, the UCDC system sent out an alarm on the cellular phone to remind the participant to measure their blood glucose, blood pressure twice a day (before breakfast and bedtime) and body weight once a day (before breakfast). The Anycheck device attached to their cellular phone conducted the glucose measurements and automatically sent the results to a central study database. As soon as**	**3 months**	**To investigate the effectiveness and applicability of the Ubiquitous Chronic Disease Care (UCDC) system using cellular phones and the internet for overweight patients with both Type 2 diabetes and hypertension**	**After 12 weeks, there were significant improvements in HbA1c in the intervention group compared with the control group. A significant reduction in systolic and diastolic blood pressure was observed, as well as improvements in total cholesterol, low-density lipoprotein cholesterol and triglyceride levels in the intervention group. There was a significant increase in adiponectin levels in the intervention group compared with the control group, although high-sensitivity C-reactive protein and interleukin-6 levels did not change in either group.**

			**participants transmitted their glucose measurement through their cellular phones, they immediately received messages of encouragement, reminders, and recommendations. Second, the UCDC system automatically recorded participant's exercise time using the SMS which was predefined according to each patient's daily schedule. Participants replied via an automatic answer system whether or not they exercised. Third, participants received information via SMS three times a day regarding healthy diet and exercise methods, along with general information about diabetes, hypertension, and obesity. Furthermore, using the internet website, physicians could follow participant's trends in blood glucose levels, blood pressure and body weight changes, allowing them to send individualized recommendations.**			
Zhou et al. 2016 (42)	RCT	N=100 (intervention group: n=50; control group: n=50) T1DM & T2DM chinese outpatients Age: Intervention group=53.5±12.4; control group: 55±13.1	mHealt technology/ tool: Welltang app (Shanghai Geping Information and Technique Company Ltd), patient's smartphone and glucometer.<br><br>Tool purpose/ functioning: Welltang is a smart phone-based diabetes management application for both patients and clinicians. Welltang for patients consists of 3 main parts: 1) knowledge, 2) self-management, and 3) communication. In the knowledge component, the Welltang database stores diabetic knowledge on diet, exercise, medicine, BGL, and the latest guidelines for diabetes care as summaries; it serves as a virtual educator for diabetes and a virtual endocrinologist for clinicians, facilitating the integration	3 months	To evaluate the impact of Welltang on HbA1c and to measure whether Welltang improves blood glucose, low-density lipoprotein cholesterol, weight, blood pressure, hypoglycemic events, satisfaction of patients, diabetes knowledge of patients, and self-care behaviors.	The average decrease in HbA1c was 1.95% (21 mmol/mol) in the intervention group and 0.79% (8 mmol/mol) in the control group. Measures of self-monitored blood glucose, diabetes knowledge, and self-care behaviors improved in patients in the intervention group. Eighty four percent of patients in the intervention group were satisfied with the use of Welltang. Differences in hypoglycemic events, low-density lipoprotein cholesterol, weight, and blood pressure were not statistically

	of diabetes care among existing resources. In the self-management component, the application allowed diabetes patients to enter their self-care data (blood glucose values, carbohydrate intake, medications, and other diabetes management information) on their smart phone, and these data were transferred to secure servers to generate into computer-generated logbooks. Data entered into or collected by these mobile devices are stored on central servers, from which it can be retrieved in the event of mobile device failure. Communication between patients and clinicians comprised patients receiving advice from the study team usually within the day based on their entered questions. The study team provided feedback on the BGL of patients, their target goals, and their individualized medication regimens based on the data they entered once a week or every 2 weeks. Predetermined safety thresholds and parameters for blood glucose and blood pressure were programmed in the database, in which readings outside threshold limits would automatically trigger a message to be sent to patients and notify clinicians. The database also triggers alerts for missed readings. These alerts are sent to the participants to encourage them to measure their data. Lastly, they received an electronic action plan as pre-visit summaries for physician office visits one a month.		significant.

BGL: Blood Glucose Level; BMI: Body Mass Index; BP: Blood pressure; CBT: Cognitive-behavioral treatment; CPDS: Coach portal with decision support; FBG: Fasting Blood Glucose; HbA1c: glycated haemoglobin; HRQoL: Health-related quality of life; MI: Motivational interviewing; n.e.; not specified; PC: Primary Care; RCT: Randomized Clinical Trial; SES: Socio-economic status; SMS: Short Message Service; SW: Software; TAU: Treatment as usual; T2DM: Type 2 Diabetes.
